# De-succinylation-induced accumulation of TRMT10C in the nucleus plays a detrimental role in coronary microembolization via its m1A modification function

**DOI:** 10.7150/ijbs.107965

**Published:** 2025-04-13

**Authors:** Chen-Kai Hu, Wan-Zhong Huang, Lei He, Chen Chang, Yan-Ling Ren, Ri-Xin Dai, Qiang Wu, Qiang Su

**Affiliations:** 1 Department of Cardiology, the Second Affiliated Hospital, Jiangxi Medical College, Nanchang University, No. 1, Minde Road, Nanchang, Jiangxi Province, China.; 2 Department of Cardiology, Jiangbin Hospital of Guangxi Zhuang Autonomous Region, No. 85 Hedi Road, Nanning, Guangxi, 530021, China.; 3 Department of Cardiology, Affiliated Hospital of Guilin Medical University, No. 15, Lequn Road, Guilin, Guangxi, 541001, China.; 4 Senior Department of Cardiology, the Sixth Medical Center, Chinese PLA General Hospital, Beijing 100048, China.

**Keywords:** coronary microembolization, succinylation, nuclear localization, m1A modification, TRMT10C, inflammation, mitophagy

## Abstract

Coronary microembolization (CME) refers to embolism in the coronary microcirculation. This study showed a reduction in succinyl transferase (CPT1A) and the succinylation substrate (succinyl-CoA) in cardiomyocytes in CME models, suppressing the succinylation of the mitochondrially localized protein TRMT10C. Suppression of succinylation promotes KPNA4 recognition of two nuclear localization signals (NLSs), KAKR and KKK(X)_10_KVKK, in TRMT10C, which induces the transport of TRMT10C from the cytoplasm to the nucleus rather than to the mitochondria. Nuclear TRMT10C induces YTHDF2-mediated decay of TAFAZZIN and NLRX1 through m1A modifications. The reduction in TAFAZZIN and NLRX1 is associated with multiple detrimental effects, such as inflammation mediated by NF-κB and NLRP3, reactive oxygen species (ROS) production, and suppression of mitophagy. TRMT10C knockdown suppressed the accumulation of TRMT10C in the nucleus. It restored NLRX1 and TAFAZZIN protein levels in cardiomyocytes under hypoxia. However, the deficiency of TRMT10C in the mitochondria did not improve—or even worsened—with TRMT10C knockdown. Inducing TRMT10C succinylation via CPT1A overexpression led to the redistribution of TRMT10C to the mitochondria rather than the nucleus, which is likely a better approach for improving cardiomyocyte function under hypoxia than direct TRMT10C knockdown. This study reveals a novel pathological mechanism underlying CME and suggests potential therapeutic targets for this disease.

## Introduction

Coronary microembolization (CME) refers to an embolism in the coronary microcirculation and a series of complications caused by myocardial microinfarction. Some CME events occur spontaneously due to the rupture of coronary atherosclerotic plaques, with microemboli ultimately becoming lodged in the coronary microcirculation. Microemboli can also be generated during percutaneous coronary interventions (PCI) and thrombolytic therapy 1. CME leads to "no-reflow," "slow-flow," and microcirculatory dysfunction, resulting in regional myocardial ischemia. However, unlike acute coronary artery embolism, the clinical presentation of CME is usually subtle or nonspecific in the early stages. In most cases, CME is detected using intravascular ultrasonography, magnetic resonance imaging, or combined imaging examinations. Increasing evidence has shown that the damage caused by CME is progressive and chronic. CME results in long-term myocardial structural and functional damage and is associated with multiple adverse cardiac events, including reduced coronary reserve, myocardial systolic dysfunction, arrhythmia, and heart failure 2. However, the mechanisms underlying CME-induced myocardial injury have not yet been fully elucidated. Based on prior experience in coronary artery disease, it is recommended that patients with CME receive thrombolytic agents, nitroglycerin, calcium channel blockers, or platelet GPIIb/IIIa receptor inhibitors to alleviate cardiac events. More specific treatment options for CME are needed following further clarification of its pathological mechanisms.

The tricarboxylic acid (TCA) cycle, a key process in oxygen-dependent glucose catabolism, is vulnerable to hypoxia caused by ischemia. Succinylation, a post-translational modification (PTM), refers to the transfer of succinyl groups from succinyl-CoA to lysine residues in proteins. Succinyl-CoA is primarily derived from the TCA cycle 3. Therefore, succinylation is presumed to be influenced by hypoxia resulting from myocardial ischemia in CME. Protein succinylation plays an important role in regulating protein activity, stability, cellular localization, and protein-protein interactions 4-6. It is also a key pathway involved in modulating protein function and signaling in response to metabolic changes 4, 6. KAT2A and CPT1A are succinyl transferases that promote protein succinylation by transferring succinyl groups from succinyl-CoA to lysine residues. Succinylation is reversible. Sirtuin5 (Sirt5) and Sirt7 catalyze the removal of succinyl groups from proteins. Succinylation has also been linked to ischemia/reperfusion-induced cardiac injury 7. However, its role in CME remains unclear.

PTMs affect multiple characteristics of proteins, such as their subcellular localization. Proteins that function in the nucleus or mitochondria must be transported to specific locations after being produced by the endoplasmic reticulum in the cytoplasm. The transport of newly synthesized protein from the cytoplasm to the nucleus is mediated by nuclear import receptors, primarily consisting of KPNA family proteins. Members of this family possess highly conserved structural domains that recognize nuclear localization signals (NLS) in cargo proteins. Classical NLSs generally contain three or more positively charged amino acids, such as arginine (R) or lysine (K) 8. Characteristic motifs include K(K/R)X(K/R) and R/K(X)_10-12_KRXK, where X represents any amino acid. KPNA family proteins also bind to importin-β, which interacts with the nuclear pore complex to facilitate the transport of KPNA and its cargo protein across the nuclear membrane 8, 9. KPNA-mediated nuclear localization is influenced by the PTMs of cargo proteins. PTMs likely regulate the interactions between KPNA family proteins and their cargo. For example, the nuclear localization of many well-known transcription factors is precisely regulated by phosphorylation and dephosphorylation, which affects their interactions with KPNA proteins 10.

Unlike PTMs, which affect protein characteristics, post-transcriptional regulation refers to various RNA modifications. N1-methyladenosine (m1A) is a widespread RNA modification found in tRNA, rRNA, and mRNA. m1A at the N1 position of adenine introduces a positive charge, which likely alters RNA structure and its interactions with proteins 11. Similar to N6-methyladenosine (m6A), m1A is formed by methyltransferases and removed by demethylases. Several m1A methyltransferases have been identified, including TRMT10C, TRMT61B, TRMT6, and TRMT61A. m1A-modified mRNA sites are recognized by m1A reader proteins, including YTHDF1, YTHDF2, and YTHDF3. These readers regulate mRNA stability and translation, thereby influencing the final gene expression 12.

Numerous pieces of evidence suggest that autophagy supports cellular homeostasis after myocardial ischemia by degrading damaged organelles and proteins 13, 14. Mitophagy, a specialized form of autophagy, is regulated by the classical PINK1-Parkin signaling pathway as well as other non-classical signals. During mitophagy, damaged mitochondria are labeled, enclosed by autophagosomes, and ultimately degraded within lysosomes 15. By removing dysfunctional mitochondria, mitophagy effectively suppresses apoptotic signals triggered by mitochondrial damage. Studies have shown that activation of mitophagy considerably inhibits ischemia-induced cardiomyocyte death and alleviates myocardial dysfunction 15, 16.

TRMT10C is an N(1)-methyltransferase primarily located in the mitochondria, with small amounts found in other cellular compartments 17. In pilot studies, we observed an abnormally increased accumulation of TRMT10C in the nucleus in both *in vivo* and *in vitro* CME models, along with a corresponding decrease in the mitochondrial TRMT10C*.* Given that the amount of mRNA in the nucleus far exceeds that in the mitochondrion, the translocation of TRMT10C to the nucleus is presumed to markedly enhance its role as an m1A writer compared to its function within the mitochondrion. However, the biological and pathological consequences of TRMT10C translocation to the nucleus remain poorly understood. This study aimed to elucidate the mechanism underlying TRMT10C nuclear translocation and to determine its downstream effects on the pathogenesis of CME.

## Materials and methods

### Animals and treatments

Sprague-Dawley rats (male; age: 6-8 weeks old; weight: 180-220 g) and wild-type (WT) C57BL/6 mice (age: 5-6 weeks; weight: 18-22 g) were obtained from Zhejiang Vital River Laboratory Animal Technology Co., Ltd. (Zhejiang, China). All animals were treated and cared for in accordance with the Guide for the Care and Use of Laboratory Animals (National Institutes of Health, Revised 2011), and all experimental procedures were approved by the Animal Care and Use Committee of Guilin Medical University (approval number: GLMC-IACUC-2021028). Animals were randomly allocated to each group, and the study was performed using a blinded method during the analysis of all animal indicators. None of the animals included in this study had congenital disabilities or congenital diseases. Animals exhibiting severe vomiting or diarrhea, difficulty breathing, central nervous system suppression, seizures, or repetitive self-harm after modeling were excluded from the study.

To establish CME models, microspheres with diameters of 45 μm (for rats) and 10 μm (for mice) (Polysciences Inc., USA) were suspended in physiological saline and immediately injected into the left ventricle via the cardiac apex. These microparticles were used to create physical embolisms in the distal coronary vasculature. Animals in the sham group received an equal volume of physiological saline. Echocardiography was used to measure left ventricular ejection fraction (LVEF) and left ventricular short axis shortening rate (LVFS). All animals were euthanized 3 days after modeling. Hearts were harvested and prepared for histopathological analysis, including hematoxylin and eosin (HE) staining and immunohistochemistry (IHC).

TRMT10C^flox/flox^ C57BL6 mice were generated using the CRISPR-Cas9 system. *TRMT10C* (National Center for Biotechnology Information [NCBI] Reference Sequence: NM_029092.4) is located on mouse chromosome 16 and contains two exons. Exon 2 was selected as the conditional knockout region (cKO) region, and its deletion resulted in the loss of function of the *TRMT10C* gene. LoxP sequences were inserted into the introns flanking exon 2 of TRMT10C. Tg(ACTA1-Cre) mice express the Cre recombinase under the control of the human alpha-skeletal actin promoter, which is active in both cardiac and skeletal muscle tissues. TRMT10C^flox/flox^ C57BL6 mice were crossed with Tg(ACTA1-Cre) mice to generate Cre^-^-TRMT10C^flox/flox^ and Cre^+^-TRMT10C^flox/flox^ mice. Knockout (KO) of TRMT10C in heart tissues of Cre^+^-TRMT10C^flox/flox^ mice was verified using polymerase chain reaction (PCR), western blotting, and IHC assays. Cre^-^-TRMT10C^flox/flox^ mice served as controls.

### HE staining and IHC

The specimens were fixed in 4% paraformaldehyde for 24 h and subjected to standard dehydration, waxing, and embedding procedures. Paraffin-embedded tissues were sectioned into 4 µm-thick slices and stained with HE using the standard method. For IHC, sections were blocked with 5% normal goat serum and incubated overnight at 4 °C with monoclonal antibodies against TRMT10C (1:100; Santa Cruz Biotechnology, Shanghai, China), TAFAZZIN (1:100; Abcam, Shanghai, China), and NLRX1 (1:100; Proteintech, Wuhan, China). The sections were then treated with a secondary biotin-conjugated antibody (Thermo, Waltham, MA, USA) at 37°C for 1 h, followed by staining with hematoxylin at 37°C for 1 min.

### Cell culture and treatments

Human and rat cardiomyocytes (AC16 and H9c2 cells, respectively) were cultured in Dulbecco's Modified Eagle Medium and Ham's F12 nutrient mixture** (**DMEM/F12) medium (Hyclone, UT, USA), supplemented with 10% fetal bovine serum and 50 U/mL penicillin and streptomycin. The cells were then transferred to hypoxic conditions (2% O_2_, 5% CO_2,_ and 93% N_2_) to mimic the ischemic state *in vivo*. It should be noted that the incubation time for cardiomyocytes under hypoxic conditions typically ranges from 2 to 24 h for most *in vitro* studies on acute coronary artery embolism. However, the exposure time to hypoxic conditions is generally longer for regional cardiomyocytes in CME than in acute coronary artery embolism in clinical settings. Therefore, for CME studies *in vitro*, the exposure time to hypoxic conditions should exceed 48 h. AC16 and H9c2 cells were collected for a series of experiments after incubation under hypoxic conditions for 48 h. To determine the autophagy/mitophagy flux, the lysosomal inhibitor bafilomycin A1 (BafA1, 20 nM) was applied for 4 h.

For the loss- and gain-of-function study, AC16 and H9c2 cells were transfected with the indicated siRNAs (GenePharma, Suzhou, China; the sequences are shown in Table S1), overexpression vectors (GenePharma), and corresponding negative controls (GenePharma) using the LipofectamineTM 2000 transfection kit (Invitrogen, USA).

### Western blot detection

Total protein in tissues and cells was extracted using radioimmunoprecipitation (RIPA) buffer supplemented with a protease inhibitor cocktail (Beyotime Institute of Biotechnology, Shanghai, China). Heart tissues were subjected to ultrasonication at 4°C to improve protein extraction. Proteins in the cell fractions, including mitochondria, cytoplasm (excluding mitochondria), and nuclei, were extracted using the Mitochondria Isolation Kit (MedChemExpress, Shanghai, China) and NE-PER Nuclear Extraction Reagents (Thermo Fisher Scientific, Shanghai, China) according to the manufacturer's instructions. Briefly, cells were homogenized for 10-30 min and centrifuged (600 × g, 4°C) for 10 min. Centrifugation removed nuclei, cell fragments, and non-lysed cells. Further centrifugation (11000 g for 10 min, 12000 g for 10 min, and 3500 × g for 10 min) separated mitochondria from the cytoplasm. A lysis buffer was used to extract proteins from the mitochondria. To extract high-purity protein from the nucleus, cells were mixed with the reagents in the nuclear extraction kit and centrifuged (16000 g, 4°C) for 10 min. The total protein and total protein from the cell fractions were collected for western blotting using standard procedures. Briefly, proteins (30 μg) were separated by sodium dodecyl sulfate-polyacrylamide gel electrophoresis, electrophoretically transferred to a nitrocellulose membrane, and probed with primary antibodies (Supplemental Table 2) and a horseradish peroxidase-conjugated secondary antibody.

### Proteomic experiments and quantification of succinylated peptides

The cells were mixed with RIPA assay buffer (Thermo Fisher Scientific) containing a protease inhibitor cocktail and phenylmethanesulfonyl fluoride. Lysates were clarified by centrifugation at 20,000 × g for 15 min, and the resuspended protein was subjected to tryptic digestion, desalting, and Tandem Mass Tag labeling. Succinylated peptides were enriched using an anti-succinyl-lysine antibody conjugated to agarose beads (PTMScan #13764; Cell Signaling Technologies) and eluted from the antibody-bead conjugates in 0.1% trifluoroacetic acid. The peptides were analyzed using nanoflow liquid chromatography-tandem mass spectrometry coupled with Q-Exactive mass spectrometry (Thermo) at the Aksomics company (Shanghai, China).

### Bioinformatics

Gene Ontology (GO) enrichment analysis was performed using DAVID Bioinformatics Resources version 6.8. The gene and protein lists were uploaded to the database to identify the most overrepresented biological processes and molecular functions correlated with the gene and protein lists. The significance of the enrichment terms was measured by calculating the *p*-value, with the threshold for significance set at *p* < 0.05 for each pathway.

### Protein structure modeling

Structural modeling was performed using the SWISS-Model Server, and multiple sequence alignments were performed using the PROMALS3D Server. Model and figure preparations were performed using Discovery Studio v4.1 (Accelrys), as previously reported.

### Immunofluorescence (IF)

Slides were fixed in 4% paraformaldehyde for 20 min and blocked with normal goat serum for 20 min at room temperature. Primary antibodies (Supplemental Table 2) were added to the cells and incubated at 4℃. Cells were then treated with secondary antibodies at 37℃ for 40 min and counterstained with 4',6-diamidino-2-phenylindole (DAPI) before being observed under a confocal microscope (Leica, TCS-SP5) using standard excitation filters. MitoMark Red and Green probes (Beyotime) were added to the cells after 30 min of incubation, and the cells were observed again under the confocal microscope.

### Plasmid construction and mutagenesis assays

Human and rat TRMT10C (NCBI reference sequences: NM_017819.4 and NM_001008337.1) were constructed by PCR amplification and subcloned into the pcDNA3.1-Flag expression vector (Invitrogen). A multipoint mutagenesis kit (Zeye Biological Company, Shanghai, China) was used to mutate specific lysine (K) residues of TRMT10C to glutamine (Q). These mutants included human Flag-TRMT10C K173Q (M1), Flag-TRMT10C K325Q (M2), Flag-TRMT10C K173Q and K325Q (M3), and rat Flag-TRMT10C K325Q (M2). All constructs were confirmed by DNA sequencing.

### Co-immunoprecipitations

Cardiomyocytes were transfected with pcDNA3.1-Flag-TRMT10C, pcDNA3.1-His-CPT1A, and pcDNA3.1-Myc-SIRT5 expression vectors to detect a change in the Succ level on Flag-TRMT10C protein. In addition, cardiomyocytes were transfected with WT and mutant vectors of Flag-TRMT10C to confirm protein interactions with KPNA2, KPNA3, and KPNA4. Cell lysates were immunoprecipitated using anti-FLAG, anti-TRMT10C, anti-PINK1, and anti-ASC antibodies. Immunoprecipitates were analyzed by western blotting using the primary antibodies listed in Table S2.

### Measurements of succinyl-CoA, reactive oxygen species (ROS), interleukin (IL)-1β, and tumor necrosis factor (TNF)-α

Succinyl-CoA in cells was measured using a succinyl-CoA detection kit (Ruixinbio, Shanghai, China) following the manufacturer's instructions. Intracellular ROS levels were evaluated using 2′,7′-dichlorofluorescin diacetate (DCFH-DA) (Beyotime Biotechnology). DCFH-DA forms the fluorescent compound dichlorofluorescein in the presence of ROS. Fluorescence was detected using flow cytometry (Fortessa, BD Biosciences). IL-1β and TNF-α released from cardiomyocytes into the culture medium were assessed using enzyme-linked immunosorbent assay detection kits (Sigma-Aldrich). The experiments were performed according to the manufacturer's instructions.

### Cell viability, proliferation, and apoptosis assays

Cell viability was assessed using the Cell Counting Kit 8 (CCK-8) (MedChemExpress, Shanghai, China). The solution from the kit was added to the cells, followed by absorbance detection at 450 nm using a microplate reader (RNE-90002 Microplate Reader, Reagent Technology Co., LTD, Shenzhen, China). Absorbance was directly proportional to cell viability. Cell proliferation was evaluated using 5-ethynyl-2′-deoxyuridine (EdU) staining. EdU staining was performed using the BeyoClick™ EdU Cell Proliferation Kit (Beyotime) according to the manufacturer's instructions. To evaluate the cell apoptosis rate, cardiomyocytes were trypsinized, incubated with Annexin V-fluorescein isothiocyanate (FITC) and propidium iodide (PI), and analyzed by flow cytometry using FlowJo software. The green fluorescence of FITC in the cells was detected in fluorescence channel 1 with a 525-nm bandpass filter, while the red fluorescence of PI in the cells was detected in fluorescence channel 2 with a 615-nm bandpass filter. After fluorescence analysis, the software generated a scatter plot with the FITC parameter as the horizontal axis and the PI parameter as the vertical axis. Cells in the areas of Annexin V^+^/PI^+^ and Annexin V^+^/PI^-^ were regarded as apoptotic cells. Apoptosis in tissue was assessed using the terminal uridine nick-end labeling (TUNEL) assay. When genomic DNA is broken, the exposed 3'-OH can be catalyzed by terminal deoxynucleotidyl transferase and connected to 2'-deoxyuridine 5'-triphosphate labeled with green fluorescence. Fluorescence was observed using a fluorescence microscope (Eclipse 80i; Nikon Instruments, Melville, New, USA).

### Quantitative real-time polymerase chain reaction (qRT-PCR) and RNA stability assays

Total RNA was extracted from cells subjected to different treatments using the TRIzol reagent (Invitrogen). RNA was reverse transcribed for real-time PCR amplification. Primers used in this study are listed in Table S 3. mRNA stability was analyzed after the cells were exposed to the transcription inhibitor actinomycin D (Sigma-Aldrich). Cells were harvested at 0, 2, 4, 8, and 12 h post-treatment, and qRT-PCR was performed to determine the expression of the target genes. 18S-RNA and β-actin were used as internal references.

### Luciferase reporter assay

The DNA fragments of the first exon of *TAFAZZIN* and the seventh exon of *NLRX1* were inserted downstream of the firefly luciferase of the modified pGL3-promoter vector (Promega, Madison, WI, USA). Compared to the WT vector, adenine was replaced with guanine in the three mutants (MT) of TAFAZZIN: MT1 (A_305_ to G), MT2 (A_413_ to G), and MT1/2 (both A_305_ to G and A_413_ to G), and in the three MTs of NLRX1: MT1 (A_2423_ to G), MT2 (A_2561_ to G), and MT3 (both A_2423_ to G and A_2561_ to G). WT and MT vectors were transfected into AC16 cells alone or in combination with TRMT10C and YTHDF2 siRNA or TRMT10C expression vectors using Lipofectamine 2000 (Invitrogen). Cells were harvested at 24 h, and the activity of firefly luciferase was normalized to that of Renilla luciferase.

### m1A and m6A-dot assay

Total RNA was isolated, gradient-diluted, spotted, and crosslinked to a nylon membrane by ultraviolet irradiation. The membrane was then blocked with 5% non-fat milk in phosphate-buffered saline with tween 20 for 1 h, incubated with anti-m1A and anti-m6A antibodies overnight at 4°C, and subsequently incubated with horseradish-peroxidase-conjugated secondary antibody. The signal was visualized using an enhanced chemiluminescence detection kit. The same membrane was stained with 0.02% methylene blue in 0.3 M sodium acetate (pH 5.2) for 2 h as a loading control.

### m1A-meRIP qPCR, m6A-meRIP, and m1A-meRIP-Seq

Total RNA was isolated, and mRNA was enriched using the Dynabeads mRNA Purification Kit (Invitrogen). The mRNA was fragmented into 100-200 bp fragments using a fragmentation reagent (Ambion). A small portion of the mRNA was used as the input, while the remaining fragmented RNA was mixed with Dynabeads Protein A (Life Technologies), which was pre-linked with anti-m1A and anti-m6A antibodies. After overnight incubation in immunoprecipitation (IP) buffer (Invitrogen), mRNA was extracted using a washing buffer. The mRNA in the input and IP samples was prepared for qPCR and next-generation sequencing using the Illumina HiSeq 2500.

### RNA binding protein IP (RIP)-qPCR

Isolated mRNA was incubated with beads coated with antibodies against YTHDF1, YTHDF2, and YTHDF3 as well as with IgG as a control. The RNA-protein-magnetic bead complexes were then washed and eluted using proteinase K digestion buffer. Immunoprecipitated RNA was extracted, quantified using qPCR, and normalized to the input.

### Polysome profiling

Polysome fractionation was performed as previously described. Briefly, the cells were treated with cycloheximide (Sigma-Aldrich) for 10 min at 37°C. The cells were then harvested, lysed on ice, and loaded onto a 10-50% sucrose gradient at 36,000 rpm. Gradient fractionation was performed and continuously monitored at 254 nm using a density gradient fractionation system (Brandel, Gaithersburg, Germany). MD, USA). The collected fractions were analyzed using qRT-PCR.

### Statistical analysis

Data are presented as mean ± standard deviation (SD). The number of experimental replicates is indicated in the figure legends. Statistical analyses were performed using GraphPad Prism version 7.0.4. The unpaired t-test was used for comparisons between two groups, while analysis of variance and Tukey's test was used for comparisons between three or more groups. The *p*-value of < 0.05 was considered statistically significant.

## Results

### Succinylation modification of protein is suppressed in CME models *in vivo* and* in vitro*

**Figure 1:**
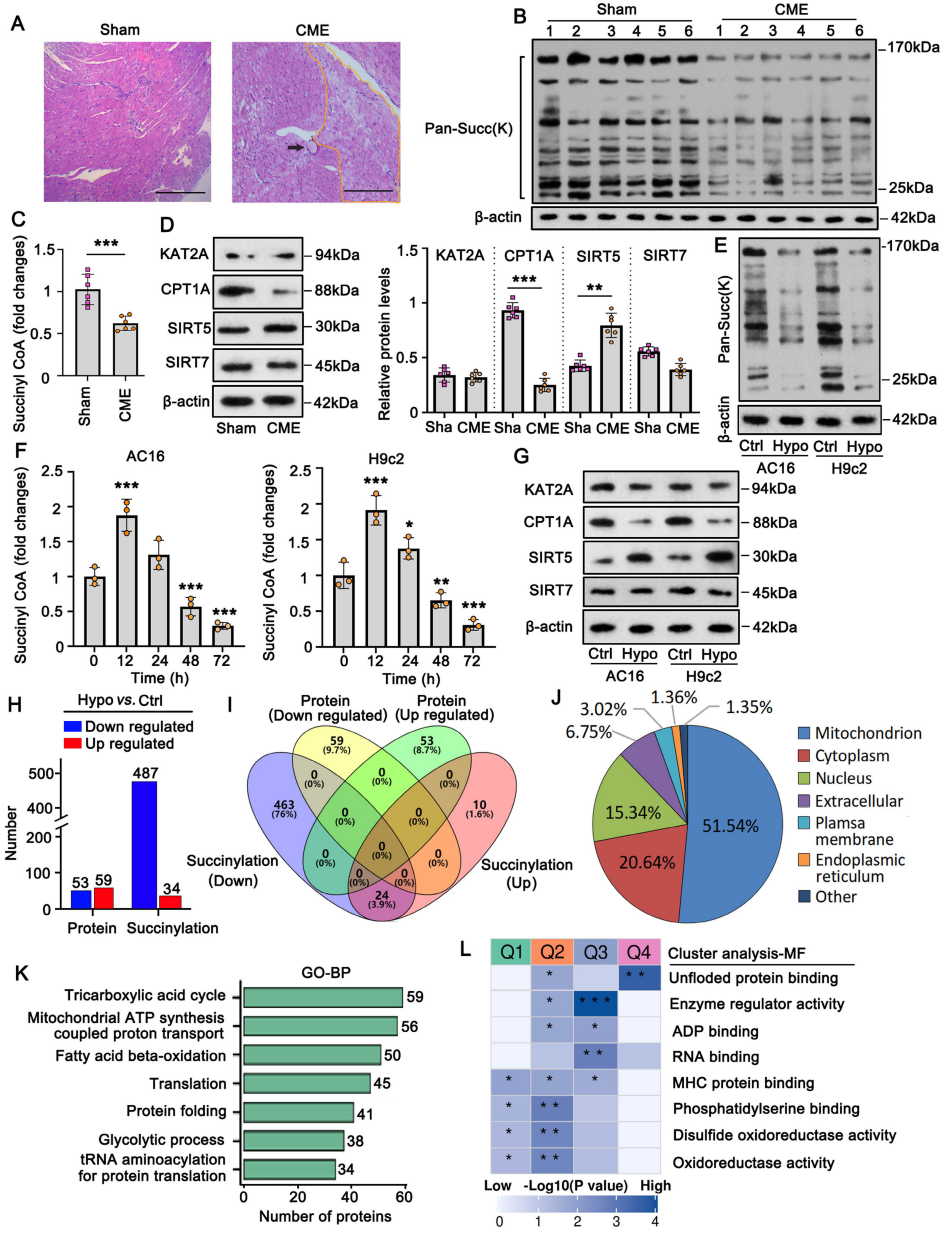
** Succinylation modification of proteins is suppressed in CME models *in vivo* and* in vitro*.** A. A rat CME model was established by injecting microspheres to occlude microvasculature. The black arrow indicates the microspheres in the heart tissues. Hematoxylin and eosin (HE) staining of heart tissues from both the sham and CME rat models. The bar in the figures represents 120 μm. B. Pan-succinylation levels in protein in both sham and CME groups were detected by western blotting with the Succ(K) antibody. C. The levels of Succinyl-CoA in the heart tissues from the sham and CME groups were detected using the detection kit. ***p<0.001 vs. Sham (n = 6). D. The protein levels of SIRT5, CPT1A, KAT2A, and SIRT7 in heart tissues from the sham and CME groups were detected by western blotting. **p<0.01 and ***p<0.001 vs. Sham (n = 6). Sha: Sham. E. Pan-succinylation levels of the protein were examined by western blotting in cardiomyocytes AC16 and H9c2 under hypoxic conditions for 48 hours. F. The levels of succinyl-CoA were examined in cardiomyocytes AC16 and H9c2 under hypoxic conditions. *p<0.05, **p<0.01 and ***p<0.001 vs. 0 hour (n = 3). G. The protein levels of SIRT5, CPT1A, KAT2A, and SIRT7 were examined in cardiomyocytes AC16 and H9c2 under hypoxia by western blot assay. H. Total protein and succinylated proteins were analyzed in AC16 cells via proteomic experiments. The numbers of proteins exhibiting downregulated and upregulated expression and succinylation are presented in a bar chart. I. Intersection analysis was conducted for proteins with downregulated and upregulated expression and succinylation. J. The distribution of proteins with changed succinylation in each cell fraction. K. Gene Ontology (GO) enrichment analysis of the biological processes associated with proteins exhibiting altered succinylation was performed. L. Cluster analysis of the biological processes related to proteins with altered succinylation is presented.

In this study, a rat CME model was established using microsphere injections. HE staining of heart tissues showed that the microspheres caused microvascular embolism and local myocardial ischemia (Figure 1A). Local myocardial ischemia results in various pathological changes, including hypoxia which impairs aerobic metabolism in mitochondria. Aerobic metabolism is not only essential for adenosine triphosphate (ATP) production but is also associated with multiple molecular biological functions, such as protein succinylation 4. However, changes in protein succinylation during CME remain unclear. In this study, we assessed pan-succinylation levels in proteins from both the sham and CME groups. The results showed that pan-succinylation levels were lower in the CME group than those in the sham group (Figure 1B). Protein succinylation depends on several succinylases, such as KAT2A and CPT1A, which add succinyl-CoA to proteins, and SIRT5 and SIRT7, which remove succinyl-CoA. Succinyl-CoA levels were significantly reduced in the myocardial tissues of the CME group (*p* <0.001; Figure 1C). In addition, the CME group exhibited decreased CPT1A (*p* <0.001; Figure 1D) and increased SIRT5 protein levels (*p* <0.01). However, KAT2A and SIRT7 protein levels did not show significant differences between the sham and CME groups.

To complement these findings, an *in vitro* CME model was established by exposing cardiomyocytes to hypoxic conditions. Pan-succinylation levels were also reduced in AC16 and H9c2 cardiomyocytes after 48 h of hypoxia (Figure 1E). Moreover, succinyl-CoA levels in AC16 and H9c2 cells increased after 12 h of hypoxic exposure (*p* <0.001; Figure 1F); however, levels decreased after 48 and 72 h of hypoxia, resulting in a reduction in succinyl-CoA (*p* <0.01 or *p* <0.001, respectively), compared to control conditions. Western blot analysis revealed that CPT1A protein levels decreased while SIRT5 protein levels increased in AC16 and H9c2 cells under hypoxic conditions (Figure 1G). KAT2A and SIRT7 protein levels did not significantly change following hypoxia exposure. In summary, both *in vivo* and *in vitro* studies suggest that reduced protein succinylation in CME is associated with a decrease in CPT1A and an increase in SIRT5 levels.

The inhibition of succinylation is likely to influence protein levels and functions. To test this hypothesis, we conducted proteomics analysis and protein succinylation modification assays in AC16 cells. Compared to the control group, the hypoxia group exhibited 53 downregulated and 59 upregulated proteins (Figure 1H and Figure S1A and B). Succinylation levels were decreased in 487 proteins and increased in 34 proteins.

However, there was no overlap between the differentially expressed proteins and those with significantly altered succinylation levels (Figure 1I). Furthermore, intersection analysis revealed that 24 proteins showed both increased and decreased succinylation levels at different sites, suggesting that succinylation levels varied by location within the same protein. Notably, the majority (51%) of proteins with altered succinylation were localized to the mitochondria (Figure 1J), followed by proteins in the cytoplasm and nucleus. To explore the molecular functions of succinylation-modified proteins, we conducted GO enrichment and cluster analyses. GO enrichment analysis showed that these proteins were primarily associated with mitochondrial functions such as the TCA cycle, ATP synthesis, and fatty acid β-oxidation (Figure 1K). Cluster analysis categorized the succinylation-modified proteins into four clusters based on molecular function (Figure 1L). Among them, Cluster Q3 had the highest *p*-value, and its proteins were primarily associated with enzyme regulatory activity and RNA binding. Within Cluster Q3, we identified the protein TRMT10C, a mitochondrial tRNA N(1)-methyltransferase, which is involved in both enzyme regulator activity and RNA binding. Based on these findings, we conducted further experiments to investigate the role of TRMT10C in CME.

### Succinylation modification affected the distribution of TRMT10C in the mitochondrion and nucleus

**Figure 2:**
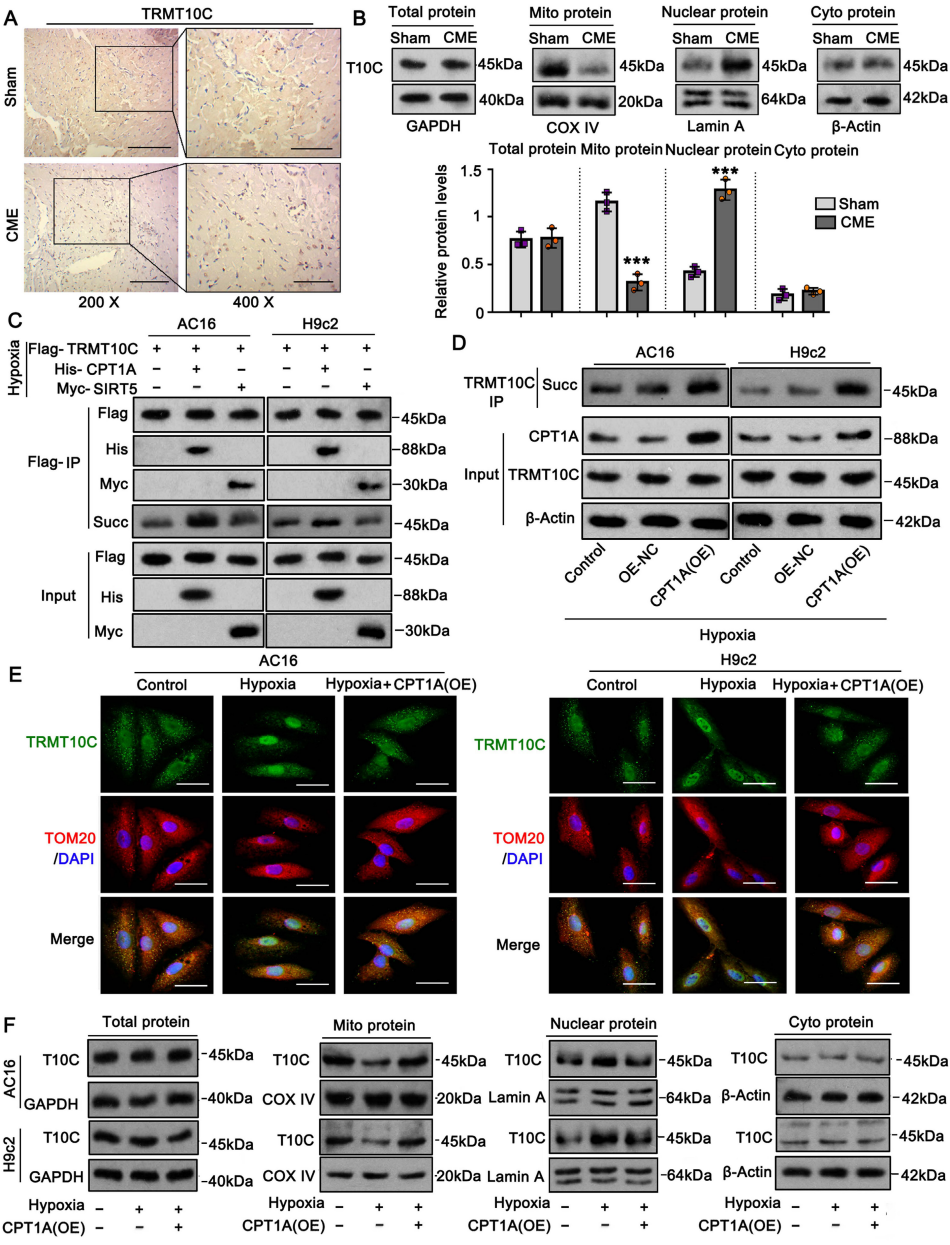
** Succinylation modification affects the distribution of TRMT10C in the mitochondrion and nucleus.** A. A rat model of CME was established by injecting microspheres to occlude the microvasculature. Immunohistochemical analysis was performed to assess the abundance and localization of TRMT10C protein in the heart tissues from both the sham and CME groups. The scale bar in the figures at 200x magnification represents 120 μm, while the scale bar at 400x magnification represents 50 μm. B. Western blot analysis was conducted to evaluate TRMT10C levels in each cell fraction from the sham and CME groups. ***p < 0.001 vs. Sham (n = 6). C. Expression vectors for Flag-TRMT10C, His-CPT1A, and Myc-SIRT5 were transfected into AC16 and H9c2 cells under hypoxic conditions. Co-immunoprecipitation (Co-IP) analysis was performed by pulling down the Flag-labeled TRMT10C protein, followed by examination of CPT1A, SIRT5, and succinylated proteins using western blotting. D. CPT1A expression vectors and control vectors were transfected into AC16 and H9c2 cells prior to hypoxic exposure. Co-IP analysis was performed to assess the succinylated proteins associated with TRMT10C. E. CPT1A expression vectors were transfected into AC16 and H9c2 cells before hypoxic exposure. An immunofluorescence (IF) assay was conducted to determine the localization of TRMT10C protein within the cells. TOM20 protein fluorescence was used to label the mitochondria, while DAPI staining was employed to visualize the nuclei. The scale bar in the figures represents 5 μm. F. Western blot analysis was performed to examine TRMT10C levels in the mitochondria, cytoplasm (excluding mitochondria), and nucleus of AC16 and H9c2 cells. Hypo: Hypoxia; T10C: TRMT10C; OE: Overexpression.

Immunohistochemical analysis revealed differences in TRMT10C protein distribution among cellular fractions between the sham and CME groups (Figure 2A). To further verify this finding, we extracted total protein as well as proteins from subcellular fractions, including mitochondria, cytoplasm (without mitochondria), and the nucleus in myocardial tissues. Western blot analysis showed no difference in total TRMT10C protein levels between the sham and CME groups (Figure 2B). However, mitochondrial TRMT10C protein levels were significantly reduced (*p* < 0.001), while nuclear TRMT10C levels were increased (*p* < 0.001) in the CME group compared to those in the sham group. Cytoplasmic TRMT10C levels remained unchanged. These findings raised the question of whether altered TRMT10C distribution was related to changes in its succinylation. To test this hypothesis, we first investigated the mechanism behind the reduced succinylation of TRMT10C in CME. As previously noted, CPT1A (a succinylase) and SIRT5 (a de-succinylase) were differentially expressed in CME. We constructed expression vectors for Flag-tagged TRMT10C, His-tagged CPT1A, and Myc-tagged SIRT5 and transfected them into AC16 and H9c2 cells. Co-IP analysis showed that His-CPT1A co-precipitated with Flag-TRMT10C to a greater extent than Myc-SIRT5 did (Figure 2C). As FLAG, His, and Myc tags do not interact with one another, this binding was solely due to the interaction between TRMT10C, CPT1A, and SIRT5. These results suggested a stronger affinity between TRMT10C and CPT1A than that between TRMT10C and SIRT5. Moreover, transfection with His-CPT1A markedly increased the succinylation level of Flag-TRMT10C, while Myc-SIRT5 transfection reduced its succinylation in H9c2 cells—a weaker effect was observed in AC16 cells. These results suggest that CPT1A plays a more important role in regulating TRMT10C succinylation under CME conditions. Under hypoxia, CPT1A overexpression increased TRMT10C succinylation (Figure 2D and S2), whereas no change was observed with a negative control vector. These results suggest that CPT1A mediates TRMT10C succinylation under hypoxic conditions. IF assays were conducted to observe TRMT10C localization in AC16 and H9c2 cells (Figure 2E). TOM20 fluorescence was used to label mitochondria, and DAPI staining marked the nuclei. Under normal conditions, TRMT10C fluorescence largely overlapped with TOM20, suggesting predominant mitochondria localization. However, hypoxic conditions led to a marked shift of TRMT10C fluorescence to the nucleus. This nuclear redistribution was reversed by CPT1A overexpression, restoring TRMT10C localization to the mitochondria. We also extracted total protein and subcellular fractions from AC16 and H9c2 cells. Western blot analysis showed no change in total TRMT10C levels after hypoxia or CPT1A overexpression (Figure 2F). However, hypoxia decreased mitochondrial TRMT10C levels and increased nuclear TRMT10C levels—a redistribution reversed by CPT1A overexpression. TRMT10C was rarely detected in the cytoplasm, and its cytoplasmic level was unaffected by either hypoxia or CPT1A expression. Overall, these findings suggest that reduced CPT1A expression in CME models suppresses TRMT10C succinylation, resulting in its abnormal accumulation in the nucleus. Restoration of CPT1A levels reversed this effect, promoting the redistribution of TRMT10C back to the mitochondria.

### TRMT10C and CPT1A regulate proliferation, apoptosis, inflammatory response, and ROS levels in cardiomyocytes under hypoxia

**Figure 3:**
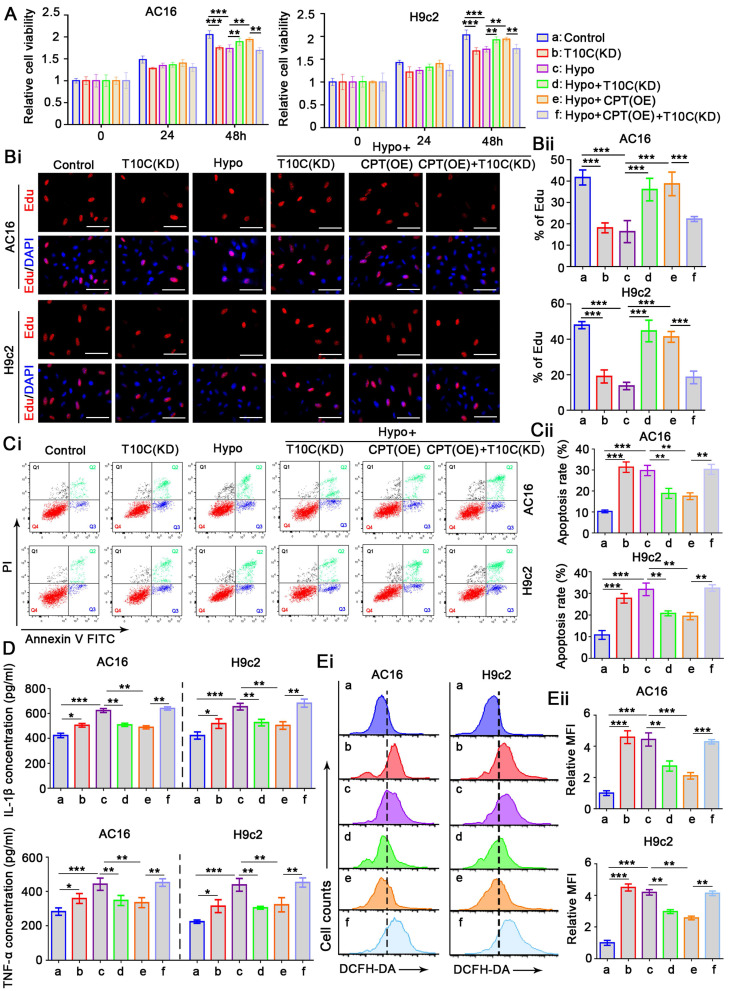
** TRMT10C and CPT1A regulate proliferation, apoptosis, inflammatory response, and ROS levels in cardiomyocytes under hypoxia.** A. siRNA targeting TRMT10C and expression vectors for CPT1A were transfected into AC16 and H9c2 cells, with some cells exposed to hypoxia. Cell viability was assessed using the CCK-8 assay. Abbreviations: T10C: TRMT10C; Hypo: Hypoxia; CPT: CPT1A; KD: Knockdown; OE: Overexpression. B. Cell proliferation was evaluated using EdU staining. Red fluorescence indicates EdU-labeled nuclei, while blue fluorescence corresponds to DAPI-stained nuclei. The cell proliferation rate is expressed as the percentage of EdU-labeled nuclei relative to DAPI-stained nuclei. The scale bar in the figures represents 10 μm. C. Cardiomyocytes were trypsinized, incubated with Annexin V-FITC and propidium iodide (PI), and analyzed by flow cytometry using FlowJo software. D. Levels of IL-1β and TNF-α in the culture medium were measured using ELISA detection kits. E. DCFH-DA reacts with reactive oxygen species (ROS) to form the fluorescent compound dichlorofluorescein. ROS levels in the cells were assessed using flow cytometry with the fluorescence probe DCFH-DA. For all studies, *p < 0.05, **p < 0.01, and ***p < 0.001 (n = 3).

The role of TRMT10C in cardiomyocytes remains unclear. To investigate this, we transfected siRNA to knockdown TRMT10C expression in AC16 and H9c2 cells (Figure S2). As a negative control, transfection with a non-targeting siRNA had no effect on cell viability in either cell line (data not shown). However, TRMT10C knockdown significantly impaired the viability of AC16 and H9c2 cells (*p* < 0.001; Figure 3A), suggesting that TRMT10C is required for normal cardiomyocyte function. Exposure to hypoxia for 48 h also reduced cardiomyocyte viability. Moreover, TRMT10C knockdown improved cell viability under hypoxic conditions (*p* < 0.01 vs. hypoxia group). Similarly, CPT1A overexpression enhanced cell viability during hypoxia (*p* < 0.01 vs. hypoxia group). However, TRMT10C knockdown attenuated the protective effect of CPT1A overexpression (*p* < 0.01 vs. hypoxia + CPT1A overexpression group). To evaluate cell proliferation, EdU staining was. TRMT10C knockdown suppressed proliferation in both AC16 and H9c2 cells (*p* < 0.001; Figure 3B). Hypoxia also inhibited cell proliferation (*p* < 0.001), but this suppression was partially alleviated by either TRMT10C knockdown or CPT1A overexpression (*p* < 0.001 vs. hypoxia group). However, when combined, TRMT10C knockdown and CPT1A overexpression failed to restore cell proliferation. Flow cytometry analysis revealed that TRMT10C knockdown increased apoptosis in AC16 and H9c2 cells (*p* < 0.001, Figure 3C). Similarly, apoptosis rates were elevated under hypoxic conditions (*p* < 0.001). Both TRMT10C knockdown and CPT1A overexpression reduced the apoptotic rate of cells under hypoxic conditions (*p* < 0.01 vs. hypoxia group). However, TRMT10C knockdown and CPT1A overexpression alone had no such effects. Stimulation of the inflammatory response in cardiomyocytes is an important characteristic of CME 18-20. Therefore, we investigated the effects of TRMT10C and CPT1A on the inflammatory response. TRMT10C knockdown promoted the production of IL-1β and TNF-α (*p* < 0.05, Figure 3D). Exposure to hypoxia strongly induced the production of IL-1β and TNF-α (*p* < 0.001). TRMT10C knockdown before hypoxia exposure hindered the production of IL-1β and TNF-α (*p* < 0.01 vs. hypoxia group). CPT1A overexpression also suppressed IL-1β and TNF-α production; however, this effect was abolished by TRMT10C knockdown. TRMT10C plays an important role in mitochondria. The accumulation of TRMT10C in the cell nucleus, rather than in the mitochondria, under hypoxic conditions likely disrupts mitochondrial function. Mitochondrial dysfunction is typically accompanied by an increase in ROS production. Uncontrolled ROS production is harmful to cells because it can activate multiple apoptotic signaling pathways 21. Therefore, ROS levels are important indicators of cellular status. ROS levels were measured by flow cytometry. Under normal conditions, TRMT10C knockdown significantly increased ROS levels (*p* < 0.001; Figure 3E). However, TRMT10C knockdown suppressed the hypoxia-induced increase in ROS levels (*p* < 0.01 vs. the hypoxia group). CPT1A overexpression also reduced ROS levels under hypoxic conditions (*p* < 0.01 or *p* < 0.001 vs. hypoxia group), but this effect was attenuated by TRMT10C knockdown (*p* < 0.01 or *p* < 0.001 vs. hypoxia + CPT1A overexpression group). Together, these findings indicate that CPT1A exerts protective effects on cardiomyocytes under hypoxia and that these protective effects are associated with the regulation of TRMT10C. In addition, the results suggest that the accumulation of TRMT10C in the nucleus is harmful to cardiomyocytes under hypoxic conditions, as reducing nuclear TRMT10C—via TRMT10C knockdown or CPT1A overexpression—alleviated cellular damage. Compared to TRMT10C knockdown, CPT1A overexpression appears to be a more effective approach for improving cardiomyocyte function under hypoxia. Notably, CPT1A overexpression has a stronger ROS-suppressive effect than TRMT10C knockdown. CPT1A overexpression promoted the redistribution of TRMT10C to the mitochondria rather than the nucleus. Although TRMT10C suppressed its accumulation in the nucleus, it did not restore TRMT10C levels in the mitochondria.

### Succinylation modification affected the connection of TRMT10C to the nuclear localization signal protein KPNA4

**Figure 4:**
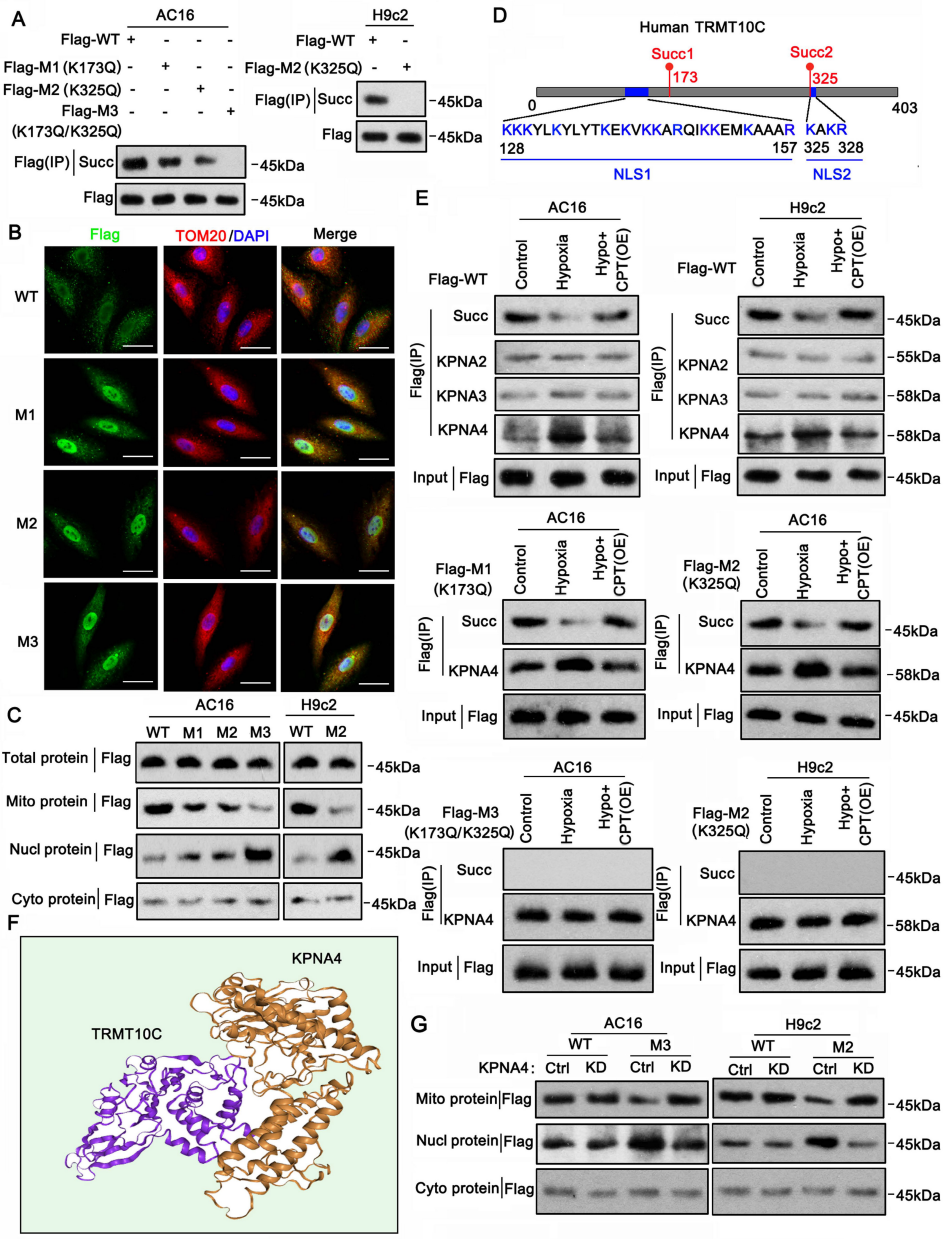
** Succinylation affects the binding of TRMT10C to the nuclear localization signal protein KPNA4.** A. This study established FLAG-tagged constructs of human and rat wild-type (WT) TRMT10C proteins, along with three mutants: human mutant 1 (K173Q, M1), human and rat mutant 2 (K325Q, M2), and the human double mutant (K173Q/K325Q, M3). Individual constructs were transfected into AC16 and H9c2 cells, and proteins were isolated by immunoprecipitation using anti-FLAG antibodies. The succinylation levels of the wild-type and mutant proteins were examined by western blotting. B. Immunofluorescence was performed in AC16 cells after transfection with the WT, M1, M2, and M3 constructs to detect the distribution of TRMT10C protein in various cell fractions. The scale bar in the figures represents 5 μm. C. Western blotting was conducted to analyze the levels of wild-type and mutant TRMT10C proteins in each cell fraction. Abbreviations: Mito: mitochondria; Nucl: nucleus; Cyto: cytoplasm. D. A schematic diagram illustrates two potential nuclear localization signals (NLS) in the human TRMT10C protein. The first NLS (NLS1) is located near the succinylation site at K173, while the second NLS (NLS2) is adjacent to another succinylation site at K325. E. AC16 and H9c2 cells were transfected with wild-type and mutant constructs prior to hypoxic exposure. CPT1A was overexpressed to restore succinylation in hypoxic cells. Co-immunoprecipitation was performed using anti-FLAG antibodies to pull down TRMT10C and its binding partners. Abbreviations: Hypo: hypoxia; OE: overexpression; CPT: CPT1A. F. Protein structure modeling demonstrates the binding of KPNA4 to TRMT10C. G. Wild-type TRMT10C proteins, as well as human M3 and rat M2 mutants, were transfected into AC16 and H9c2 cells with KPNA4 knockdown. Western blotting was conducted to detect proteins in each cell fraction. Abbreviations: Ctrl: control; KD: knockdown; Mito: mitochondria; Nucl: nucleus; Cyto: cytoplasm.

Mass spectrometric analyses revealed succinylation at K173 and K325 in the human TRMT10C protein (Figure S3). We analyzed the conservation of this protein at these two sites in humans, rats, and mice. TRMT10C lacks K173 in both rats and mice (Figure S4). However, K325 and its adjacent amino acid sequence (GTSLA**K_325_**AKR) were highly conserved among the three species (Figure S4). We investigated whether the loss of succinylation via mutations at these two sites could affect the distribution of the TRMT10C protein in cell fractions. We generated FLAG-tagged constructs of human and rat WT TRMT10C proteins and three mutants: human mutant1 (K173Q, M1), human and rat mutant2 (K325Q, M2), and human double mutant (K173Q/K325Q, M3). Each construct was transfected into AC16 and H9c2 cells, and proteins were isolated by FLAG immunoprecipitation. In AC16 cells, robust succinylation was observed in WT TRMT10C (Figure 4A). Succinylation was reduced in both M1 and M2, with a greater reduction in M2 than in M1. Succinylation was not detected in the double mutant M3. As TRMT10C lacks K173 in rats, we only transfected WT and M2 constructors into H9c2 cells. Succinylation was detected in WT-transfected cells but not in M2 H9c2 cells. We conducted IF in AC16 cells after transfection with WT, M1, M2, and M3 constructs to detect the distribution of the TRMT10C protein in the cell fractions. TOM20 fluorescence was used to mark mitochondria (Figure 4B), and DAPI staining was used to visualize cell nuclei. In WT-transfected cells, FLAG and TOM20 fluorescence overlapped, indicating that TRMT10C was primarily located in the mitochondria. However, after transfection with M1, M2, and M3, FLAG fluorescence accumulated in the nucleus, with a significant reduction in FLAG fluorescence in the mitochondria. Western blotting was conducted to examine WT and mutant TRMT10C proteins in each cell fraction. In AC16 cells, total FLAG-TRMT10C protein levels were similar after transfection with WT, M1, M2, and M3 constructs (Figure 4C). WT FLAG-TRMT10C accumulated in the mitochondria, whereas the levels of M1 and M2 in the mitochondria were lower than those of the WT. M3 showed the lowest mitochondrial localization among all FLAG-TRMT10C proteins. The amount of WT FLAG-TRMT10C in the nucleus was very low and less than that of all mutants. Among the mutants, M3 had the highest level in the nucleus. The levels of WT and mutant proteins in the cytoplasm (after removing mitochondria) were low and showed no significant differences. In H9c2 cells, total protein levels of WT and M2 were similar. The WT protein predominantly accumulated in the mitochondria, with a minimal presence in the nucleus. In contrast, M2 protein accumulated in the nucleus, with little protein in the mitochondria. The amounts of WT and mutant proteins in the cytoplasm (after removing mitochondria) were low and showed no significant differences.

Almost all proteins are generated in the endoplasmic reticulum and Golgi apparatus in the cytoplasm. Proteins are transported to various organelles through complex transport systems. The transport of proteins from the cytoplasm to the nucleus is dependent on the NLS, although the mechanism of protein transport from the cytoplasm to the mitochondria is not clearly understood. We identified two potential NLS in TRMT10C (Figure 4D). The first NLS (NLS1) is located close to the succinylation site at K173, and the second NLS (NLS2) is located adjacent to another succinylation site at K325. NLS-mediated transport depends on KPNA family proteins, such as KPNA2, KPNA3, and KPNA4. Next, we explored whether TRMT10C can bind to KPNA family proteins and whether this binding is affected by the succinylation of TRMT10C. AC16 and H9c2 cells were transfected with the WT or mutant constructs before exposure to hypoxia. CPT1A overexpression restores succinylation in hypoxic cells. Co-immunoprecipitation was performed using an anti-FLAG antibody to pull down TRMT10C and its binding proteins. The results indicated a weak connection between TRMT10C and KPNA2, KPNA3, and KPNA4 under normal conditions (Figure 4E). However, under hypoxic conditions, the enrichment of KPNA4 in the TRMT10C complex increased. CPT1A overexpression reduced KPNA4 binding to TRMT10C under hypoxic conditions. Among the KPNA family proteins, KPNA4 showed the strongest interaction with TRMT10C under hypoxia. In addition, TRMT10C was predicted to bind KPNA4 directly (Figure 4F). We further examined the interaction between KPNA4 and the TRMT10C mutants.

When M1 was transfected into AC16 cells, exposure to hypoxia reduced TRMT10C succinylation but increased KPNA4 enrichment. CPT1A overexpression restored succinylation and reduced KPNA4 enrichment. Similarly, in M2-transfected AC16 cells, hypoxia decreased succinylation and increased KPNA4 binding, which was reversed by CPT1A overexpression. However, in H9c2 cells, succinylation was not detected in rat M2 TRMT10C, regardless of hypoxia or CPT1A overexpression. KPNA4 showed a high affinity for rat M2 TRMT10C in H9c2 cells, and this affinity was not influenced by hypoxia or CPT1A overexpression. Succinylation was also not detected in human M3 TRMT10C in AC16 cells. KPNA4 showed a similarly high affinity for human M3, unaffected by hypoxia or CPT1A overexpression. These results suggest that blocking the succinylation of TRMT10C via K amino acid mutation enhances its binding to KPNA4. To confirm KPNA4's role in the nuclear transport of TRMT10C, we knocked down KPNA4 expression. WT human and rat TRMT10C accumulated in the mitochondria and were not affected by KPNA4 knockdown (Figure 4G). In contrast, human M3 and rat M2 TRMT10C proteins accumulated in the nucleus. KPNA4 knockdown reduced the nuclear levels of these proteins and increased their mitochondrial levels. Notably, KPNA4 knockdown did not markedly increase M3 or rat M2 TRMT10C protein levels in the cytoplasm. In summary, this study identified two NLSs in human TRMT10C. One of the NLS motifs (KAKR) is also present in mouse TRMT10C. The succinylation site K325 (Succ2) is located within the K325AKR motif. Another potential NLS in human TRMT10C [KKK(X)10KVKK, where X is any other amino acid] is located near the succinylation site at K173 (Succ1). Overall, succinylation at both K173 and K325 prevents the binding of KPNA4 to human TRMT10C, thereby reducing its nuclear localization.

### TRMT10C suppresses TAFAZZIN and NLRX1 expression via m1A modification

**Figure 5:**
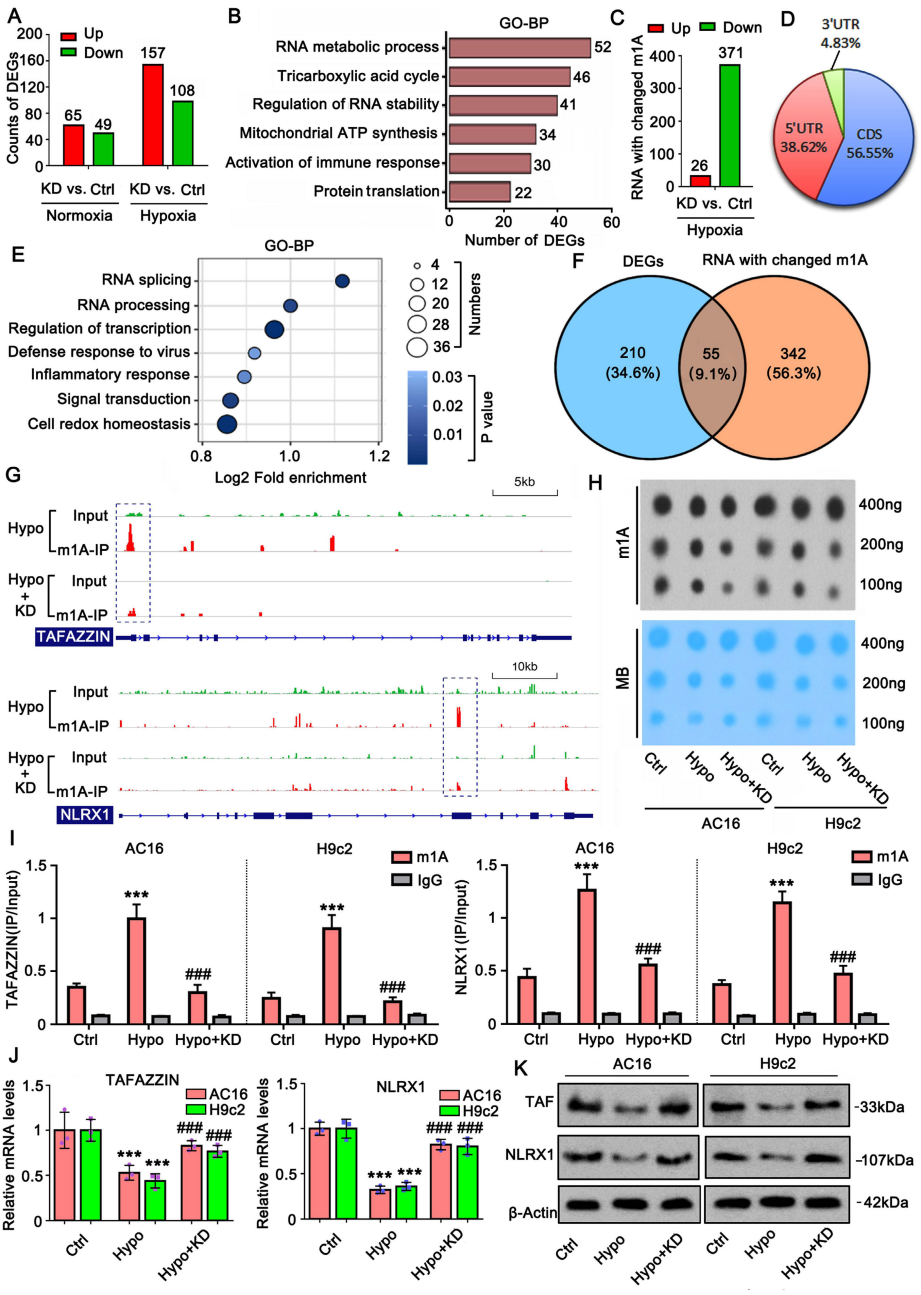
** TRMT10C suppresses TAFAZZIN and NLRX1 expression via m1A modification.** A. High-throughput sequencing was performed to evaluate changes in gene expression following TRMT10C knockdown under both normoxic and hypoxic conditions. Abbreviations: Ctrl: control; KD: TRMT10C knockdown. B. Gene Ontology (GO) enrichment analysis was conducted on the biological processes related to differentially expressed genes (DEGs). C. M1A-IP sequencing was performed to examine changes in mRNA m1A levels after TRMT10C knockdown under hypoxic conditions. D. According to M1A-IP sequencing, the distribution of m1A modifications across different regions of mRNA was analyzed. E. GO enrichment analysis was conducted for biological processes associated with mRNAs exhibiting changes in m1A modifications. F. Intersection analysis was performed between DEGs and mRNAs with altered m1A modifications. G. M1A-IP sequencing revealed a reduction in m1A levels in the mRNA from the first exon of TAFAZZIN following TRMT10C knockdown. A similar reduction in m1A levels was observed in the mRNA from the seventh exon of NLRX1 after TRMT10C knockdown. Abbreviations: Hypo: hypoxia; Ctrl: control; KD: TRMT10C knockdown. H. A dot blot assay was conducted to detect overall m1A levels in cells after exposure to hypoxia and TRMT10C knockdown. The gray value of the dot blot was used to evaluate m1A levels. The same membrane was stained with 0.02% methylene blue (MB) as a loading control. I. An M1A-IP-PCR assay was performed to examine m1A levels in both TAFAZZIN and NLRX1 mRNA in cells following hypoxic exposure and TRMT10C knockdown. ***p < 0.001 vs. Control (n = 3); ###*p* < 0.001 vs. Hypoxia group (n = 3). J and K. PCR and western blot assays were conducted to assess the mRNA and protein levels of TAFAZZIN and NLRX1 in cells. ***p < 0.001 vs. Control (n = 3); ###*p* < 0.001 vs. Hypoxia group (n = 3).

tRNA and mRNA are substrates of TRMT10C for m1A modification. Under normal conditions, TRMT10C mostly accumulates in the mitochondria, where the amount of tRNA and mRNA is relatively low compared to that in the nucleus. When TRMT10C accumulates dramatically in the cell nucleus owing to the loss of succinylation, it can access more tRNA and mRNA in the nucleus than in the mitochondria. Therefore, TRMT10C can regulate the expression of more genes in the nucleus than in the mitochondrion by acting as an 'm1A writer.' We conducted high-throughput sequencing to evaluate changes in gene expression when the distribution of TRMT10C shifted to the nucleus under hypoxia. TRMT10C knockdown in AC16 cells under normoxic conditions resulted in the upregulation of 65 genes and the downregulation of 49 genes (*p* < 0.05, FC>±2; Figure 5A).

Under hypoxic conditions, TRMT10C knockdown induced more differentially expressed genes (DEGs), including 157 upregulated and 108 downregulated genes. GO enrichment analysis of these DEGs showed that TRMT10C knockdown primarily influenced biological processes such as RNA metabolism, the TCA cycle, RNA stability, and immune response (Figure 5B). We also conducted M1A-IP-sequencing to examine changes in mRNA m1A levels after TRMT10C knockdown under hypoxic conditions. Twenty-six mRNAs showed increased m1A levels following TRMT10C knockdown (Figure 5C). However, 371 mRNAs exhibited decreased m1A levels. Most of the m1A changes (~56%) occurred in the CDS region of mRNA, followed by approximately 38% in the 5'UTR (Figure 5D). Only 4% of m1A occurred in the 3'-UTR. We explored the biological processes associated with these m1A-modified mRNAs. GO enrichment analysis revealed that the most affected processes included RNA splicing, RNA processing, transcriptional regulation, defense response to viruses, inflammatory response, signal transduction, and redox homeostasis (Figure 5E). Of course, not all m1A modifications influence mRNA or protein levels. Intersection analysis showed that 55 DEGs induced by TRMT10C knockdown under hypoxia were accompanied by altered m1A modifications (Figure 5F), suggesting that m1A modification likely contributes to the expression of these genes. Among them, we noted *TAFAZZIN* and *NLRX1*, as both are closely associated with mitochondrial function, inflammatory response, and redox homeostasis 22-24, which are known to be susceptible to the CME. M1A-IP-sequencing showed that m1A levels were reduced in the first exon of TAFAZZIN after TRMT10C knockdown (Figure 5G), while a reduction in m1A was also observed in the seventh exon of NLRX1.

Dot blot assay revealed that the overall m1A level increased under hypoxia, but this increase was suppressed by TRMT10C knockdown (Figure 5H). M1A-IP-PCR assays showed that hypoxia increased m1A levels in both TAFAZZIN and NLRX1 mRNA (*p* < 0.001, Figure 5I), while TRMT10C knockdown reversed this effect, as confirmed by PCR and western blotting. Increased m1A under hypoxia was associated with reduced mRNA and protein levels of TAFAZZIN and NLRX1 (*p* < 0.001; Figure 5J and K). TRMT10C knockdown restored the mRNA and protein levels of both genes (*p* < 0.001 vs. hypoxia group), suggesting that TRMT10C suppresses TAFAZZIN and NLRX1 levels, likely via m1A modification.

### m1A modification attenuates the stability of TAFAZZIN and NLRX1 mRNA but has no effect on the translation

**Figure 6:**
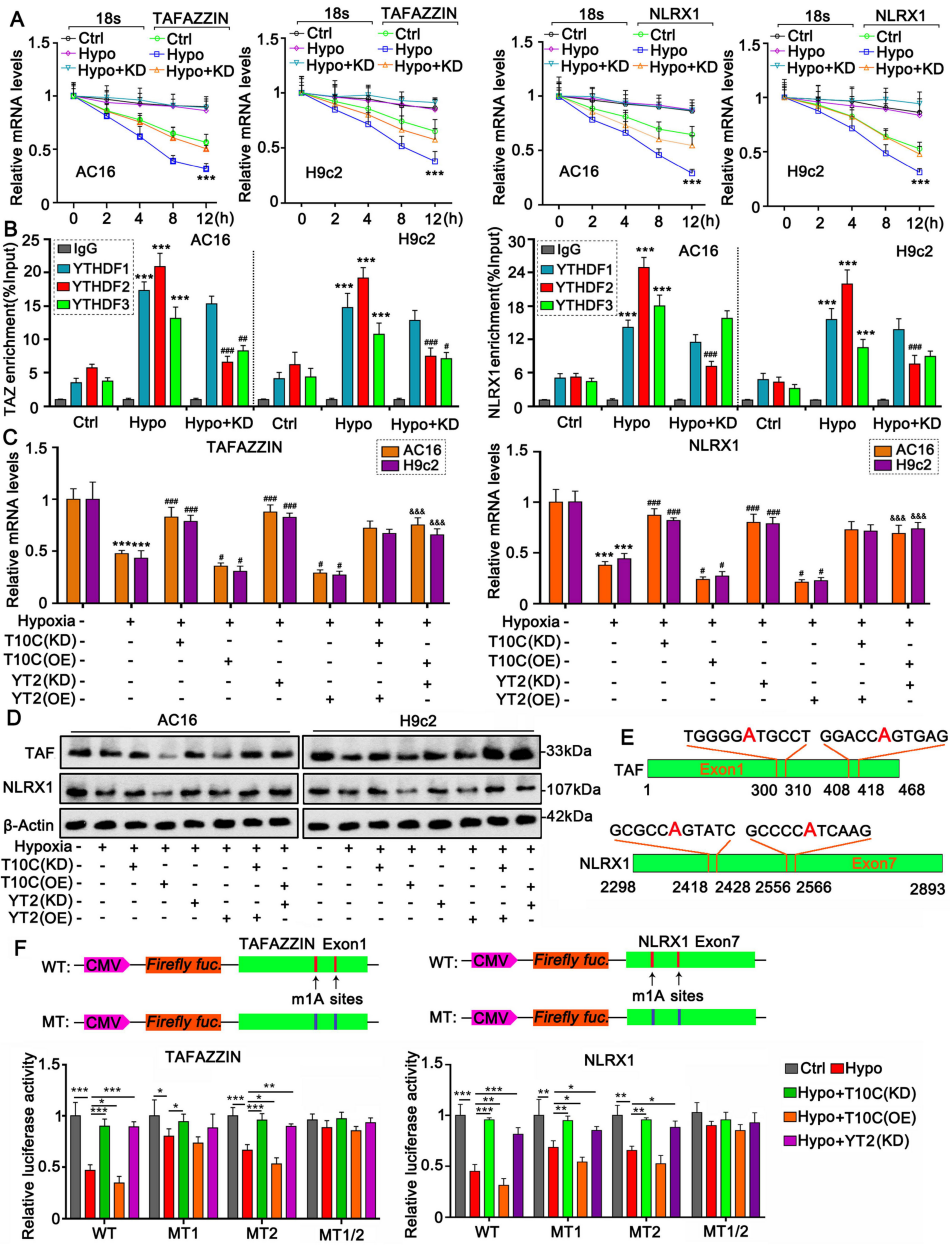
** m1A modification attenuates the stability of TAFAZZIN and NLRX1 mRNA.** A. To assess the effect of m1A modification on mRNA stability, Actinomycin D was added to the cells to inhibit the synthesis of new mRNA. PCR was performed to evaluate mRNA levels at different time points. Abbreviations: Hypo: hypoxia; Ctrl: control; KD: TRMT10C knockdown. ***p < 0.001 vs. Control (n = 3). B. A RNA immunoprecipitation (RIP) assay was conducted in cardiomyocytes following hypoxic exposure and TRMT10C knockdown. Antibodies targeting YTHDF1, YTHDF2, and YTHDF3 were used to precipitate protein-mRNA complexes. Subsequently, PCR was performed to assess mRNA levels. ***p < 0.001 vs. Control (n = 3); ##*p* < 0.01 and ###*p* < 0.001 vs. Hypoxia group (n = 3). C. TRMT10C and YTHDF2 were either overexpressed or knocked down in cardiomyocytes under hypoxic conditions. PCR was conducted to detect the mRNA levels of TAFAZZIN and NLRX1. Abbreviations: T10C: TRMT10C; YT2: YTHDF2. ***p < 0.001 vs. Control (n = 3); #*p* < 0.05 and ###*p* < 0.001 vs. Hypoxia group (n = 3); &&&*p* < 0.001 vs. Hypoxia + TRMT10C overexpression group (n = 3). D. Western blotting was performed to measure the protein levels of TAFAZZIN and NLRX1. E. The schematic diagram illustrates the m1A modification sites in TAFAZZIN and NLRX1 mRNAs. F. In the luciferase reporter assay, we constructed a wild-type (WT) vector containing the cDNA of the first exon of TAFAZZIN and the seventh exon of NLRX1. Additionally, three mutants (MT) of TAFAZZIN were generated by substituting adenine with guanine: MT1 (A_305_ to G), MT2 (A_413_ to G), and MT1/2 (both A_305_ to G and A_413_ to G). Three mutants of NLRX1 were also constructed: MT1 (A_2423_ to G), MT2 (A_2561_ to G), and MT3 (both A_2423_ to G and A_2561_ to G). Relative luciferase activity was evaluated following various cell treatments. *p < 0.05, **p < 0.01, and ***p < 0.001 (n = 3).

To determine the effect of m1A modification on mRNA stability, Actinomycin D was added to cells to block new mRNA synthesis. PCR was conducted to examine mRNA levels at different time points. After blocking mRNA synthesis, both TAFAZZIN and NLRX1 mRNA levels decreased over time (Figure 6A). The reduction in TAFAZZIN and NLRX1 mRNA levels was accelerated under hypoxic conditions (*p* < 0.001 at the 12-h time point). TRMT10C knockdown retarded the reduction in TAFAZZIN and NLRX1 mRNA levels under hypoxic conditions, indicating that TRMT10C is involved in the regulation of TAFAZZIN and NLRX1 mRNA stability under hypoxia. However, as an m1A writer, TRMT10C regulates mRNA stability through m1A readers. We conducted a RIP assay to determine which m1A readers interact with TRMT10C in the regulation of TAFAZZIN and NLRX1. Hypoxia treatment promoted the binding of m1A readers YTHDF1, YTHDF2, and YTHDF3 to TAFAZZIN and NLRX1 mRNA (*p* < 0.001, Figure 6B). TRMT10C knockdown suppressed the binding of YTHDF2 (*p* < 0.001 vs. hypoxia group) and YTHDF3 (*p* < 0.05, *p* < 0.01, vs. hypoxia group) to TAFAZZIN mRNA. In addition, TRMT10C knockdown inhibited the binding of YTHDF2 (*p* < 0.001 vs. the hypoxic group) to NLRX1 mRNA. These results indicate that TRMT10C influences the binding of YTHDF2 to both TAFAZZIN and NLRX1 mRNA. PCR and western blot assays were conducted to confirm that TRMT10C and YTHDF2 work together to regulate TAFAZZIN and NLRX1 expression. Notably, regardless of knockdown or overexpression, TRMT10C and YTHDF2 had moderate effects on the expression of TAFAZZIN and NLRX1 under normoxic conditions (Figure S5). However, both TRMT10C and YTHDF2 knockdown reversed the reduction in TAFAZZIN and NLRX1 expression under hypoxia (*p* < 0.001 vs. the hypoxia group, Figure 6C and S6). TRMT10C and YTHDF2 overexpression further lowered TAFAZZIN and NLRX1 expression under hypoxia conditions (*p* < 0.05). TRMT10C knockdown reversed the reduction in TAFAZZIN and NLRX1 expression under hypoxia, which was not influenced by YTHDF2 overexpression. Compared to TRMT10C overexpression alone, TRMT10C overexpression together with YTHDF2 knockdown reversed the reduction in TAFAZZIN and NLRX1 under hypoxia (*p* < 0.001). TAFAZZIN and NLRX1 protein levels decreased under hypoxic conditions (Figure 6D). TRMT10C overexpression further induced a reduction in TAFAZZIN and NLRX1 protein levels under hypoxic conditions. YTHDF2 knockdown attenuated the reduction in TAFAZZIN and NLRX1 protein levels under hypoxia.

As indicated by m1A-IP-sequencing, m1A levels changed at two locations: adenine (A)_305_ and A_413_ in human TAFAZZIN mRNA after TRMT10C knockdown (Figure 6E). Both genes were located within the first exon of the mRNA. m1A levels were also altered at two locations: adenine (A)_2423_ and A_2561_ in human NLRX1 mRNA after TRMT10C knockdown. Both genes are located within the seventh exon of NLRX1. A luciferase reporter assay was conducted on AC16 cells to confirm the role of adenine in the regulation of TAFAZZIN and NLRX1 (Figure 6F). We constructed the WT plasmids, in which the cDNA of the first exon of TAFAZZIN and the cDNA of the seventh exon of NLRX1 were inserted into luciferase vectors. In addition, three mutants (MT) of TAFAZZIN—MT1 (A_305_ to G), MT2 (A_413_ to G), and MT1/2 (both A_305_ to G and A_413_ to G) were generated by replacing adenine with guanine. Three mutants of NLRX1—MT1 (A_2423_ to G), MT2 (A_2561_ to G), and MT3 (both A_2423_ to G and A_2561_ to G)—were also constructed.

Luciferase activity of TAFAZZIN and NLRX1 WT was reduced under hypoxia (*p* < 0.001 vs. control), which was restored by knockdown of TRMT10C and YTHDF2 (*p* < 0.001 vs. hypoxia). TRMT10C overexpression further reduced luciferase activity under hypoxic conditions (*p* < 0.05). For MT1 (A_305_ to G) of TAFAZZIN, luciferase activity was reduced under hypoxic conditions (*p* < 0.05). TRMT10C knockdown restored luciferase activity (*p* < 0.05). However, the effects of TRMT10C overexpression and YTHDF2 knockdown on luciferase activity were marginal. Similar to WT, MT2 (A_413_ to G) showed decreased luciferase activity under hypoxic conditions (*p* < 0.001). Both TRMT10C and YTHDF2 knockdown (*p* < 0.01 or *p* < 0.001) restored luciferase activity. TRMT10C overexpression further reduced luciferase activity under hypoxic conditions (*p* < 0.05). Therefore, A_305_ is more important than A_413_ in the regulation of TAFAZZIN expression. Luciferase activity of MT1/2 cells was unaffected by the treatment. Both MT1 (A_2423_ to G) and MT2 (A_2561_ to G) of NLRX1 showed decreased luciferase activity (*p* < 0.01). Decreased luciferase activity was restored by TRMT10C and YTHDF2 knockdown (*p* < 0.05, *p* < 0.01, respectively, vs. hypoxia). TRMT10C overexpression further reduced m1A luciferase activity under hypoxic conditions. However, the luciferase activity of MT1/2 of NLRX1 was unaffected after treatment. Therefore, both A_2423_ and A_2561_ are involved in the regulation of NLRX1 expression.

Previous studies have also implicated m6A modification in the pathogenesis of multiple ischemia-induced cardiac diseases 25, 26. Using the m6A dot blot assay, we found that whole m6A levels increased in AC16 and H9c2 cells under hypoxic conditions (Figure S7A). However, TRMT10C knockdown did not affect m6A levels in hypoxic cells. Analysis of m6A-IP-sequencing showed that m6A levels in TAFAZZIN and NLRX1 mRNA also increased under hypoxia (Figure S7B). The increased m6A levels were not affected by TRMT10C knockdown. These data suggest that TRMT10C regulates TAFAZZIN, and NLRX1 is independent of m6A modification.

We investigated whether YTHDF3 is involved in the regulation of TAFAZZIN in hypoxic cells. YTHDF3 knockdown did not restore TAFAZZIN expression in AC16 and H9c2 cells under hypoxic conditions (Figure S7C). These results suggest that YTHDF3 alone does not affect TAFAZZIN expression in cardiomyocytes under hypoxic conditions. As indicated by the polysome profiling assay, the density gradient fractionation system generated a polysome profile from light to heavy fractions, including fractions with 40S, 60S, and 80S (monosomes) and polysomes (Figure S8). As these peaks did not appear to differ between treated and control cells, the treatment did not seem to have a major effect on mRNA translation. Therefore, TRMT10C-mediated m1A modification has no effect on the translation of TAFAZZIN and NLRX1.

### TAFAZZIN and NLRX1 are involved in the effects of TRMT10C on the proliferation, apoptosis, inflammatory response, and ROS in cardiomyocytes under hypoxia

**Figure 7:**
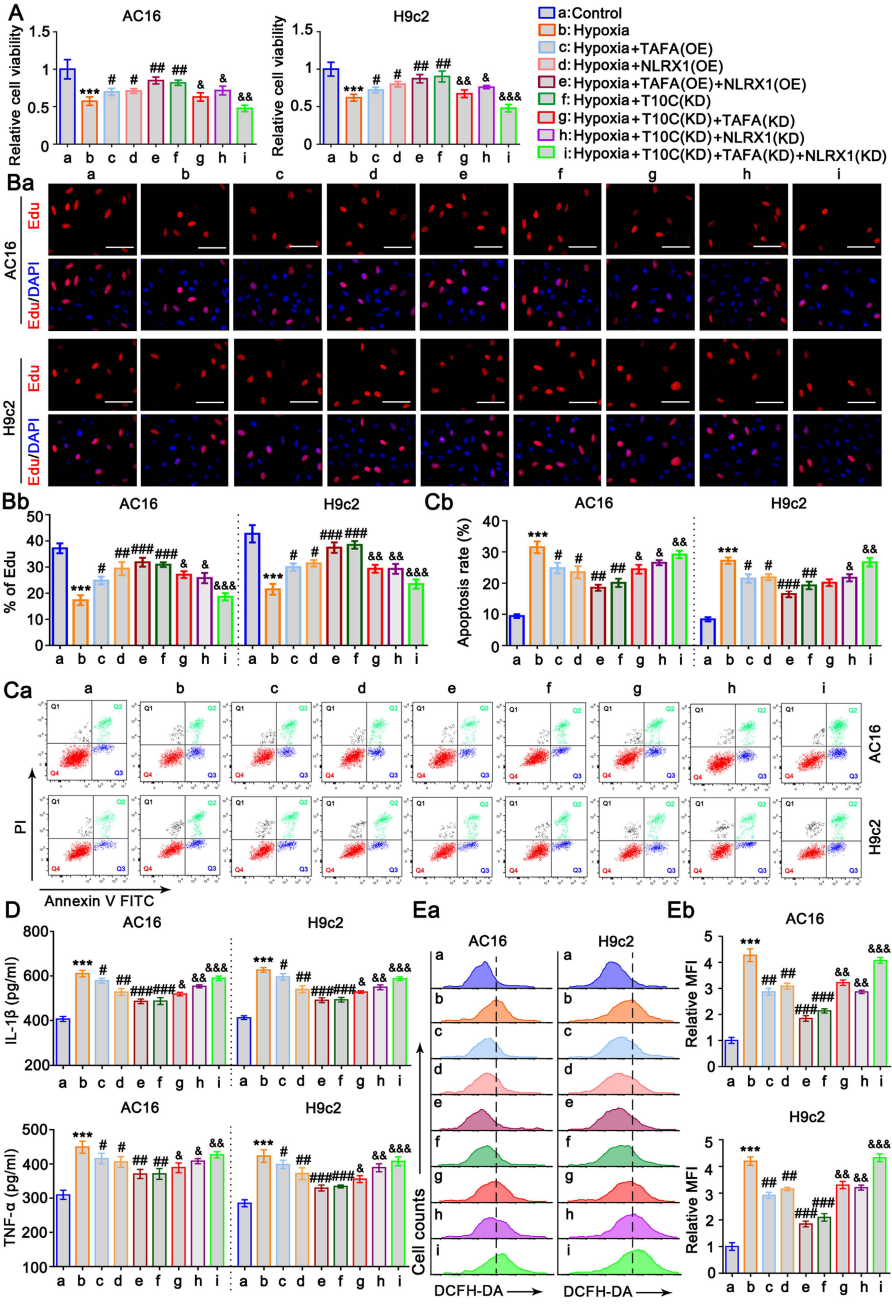
** TRMT10C influences cell proliferation, apoptosis, inflammatory response, and ROS levels in cardiomyocytes under hypoxia by regulating TAFAZZIN and NLRX1.** A. AC16 and H9c2 cells were transfected with expression vectors for TAFAZZIN and NLRX1 to prevent their reduction under hypoxic conditions. Additionally, TRMT10C was knocked down either alone or in combination with TAFAZZIN and NLRX1. Cell viability was assessed using the CCK-8 assay. Abbreviations: T10C: TRMT10C; TAFA: TAFAZZIN; KD: Knockdown; OE: Overexpression. B. Cell proliferation was evaluated using EdU staining. Red fluorescence indicates EdU-labeled nuclei, while blue fluorescence corresponds to DAPI-stained nuclei. The cell proliferation rate is expressed as the percentage of EdU-labeled nuclei relative to DAPI-stained nuclei. The scale bar in the figures represents 10 μm. C. Cardiomyocytes were trypsinized, incubated with Annexin V-FITC and propidium iodide (PI), and analyzed by flow cytometry using FlowJo software. D. Levels of IL-1β and TNF-α in the culture medium were measured using ELISA detection kits. E. Reactive oxygen species (ROS) levels in the cells were assessed using flow cytometry with the fluorescent probe DCFH-DA. For all studies, ***p < 0.001 vs. Control (n = 3); #*p* < 0.05, ##*p* < 0.01, and ###*p* < 0.001 vs. Hypoxia group (n = 3); &*p* < 0.05, &&*p* < 0.01, and &&&*p* < 0.001 vs. Hypoxia + TRMT10C knockdown group (n = 3).

AC16 and H9c2 cells were transfected with TAFAZZIN and NLRX1 expression vectors to suppress their reduction under hypoxic conditions (Figure S6). The cell viability assay showed that the elevation of TAFAZZIN and NLRX1 expression restored the viability of AC16 and H9c2 cells under hypoxia (*p* < 0.05, *p* < 0.01, respectively, vs. the hypoxia group, Figure 7A). The combination of TAFAZZIN and NLRX1 had a greater effect on cell viability than their individual effects. TRMT10C knockdown also elevated the expression of TAFAZZIN and NLRX1 and improved cell viability under hypoxic conditions. To determine if the enhanced effect of TRMT10C knockdown on cell viability was related to TAFAZZIN and NLRX1, we knocked down TAFAZZIN and NLRX1 together with TRMT10C knockdown. The enhanced effect of TRMT10C knockdown on cell viability was attenuated by TAFAZZIN and NLRX1, knockdown alone and in combination (*p* < 0.05, *p* < 0.01, or *p* < 0.001 vs. hypoxia + TRMT10C knockdown). Cell proliferation was evaluated using EdU staining. Hypoxia-suppressed cell proliferation was maintained by TAFAZZIN and NLRX1 overexpression alone (*p* < 0.05, or *p* < 0.01 vs. hypoxia group, Figure 7B) and in combination (*p* < 0.01, or *p* < 0.001 vs. hypoxia group). TRMT10C knockdown also improved cell proliferation (*p* < 0.001 vs. hypoxia group), but the improvement was impaired by TAFAZZIN and NLRX1 knockdown alone and in combination (*p* < 0.05, *p* < 0.01, or *p* < 0.001 vs. hypoxia + TRMT10C knockdown). Hypoxia-induced apoptosis was suppressed by TAFAZZIN and NLRX1 overexpression alone (*p* < 0.05, hypoxia group, Figure 7C) or in combination (*p* < 0.01, *p* < 0.001, vs. hypoxia group). TRMT10C knockdown suppressed hypoxia-induced apoptosis (*p* < 0.01 vs. hypoxia group). TAFAZZIN knockdown attenuated the anti-apoptotic effect of TRMT10C knockdown in AC16 cells (*p* < 0.05 vs. hypoxia + TRMT10C knockdown) but not in H9c2 cells. NLRX1 knockdown alone or in combination with TAFAZZIN knockdown attenuated the anti-apoptotic effects of TRMT10C knockdown in both cell types (*p* < 0.05, *p* < 0.01, and hypoxia + TRMT10C knockdown, respectively). Hypoxia-induced the production of IL-1β and TNF-α in AC16 and H9c2 cells, which was suppressed by TAFAZZIN overexpression (*p* < 0.05 vs. hypoxia group, Figure 7D), NLRX1 overexpression (*p* < 0.05 or *p* < 0.01 vs. hypoxia group), as well as the overexpression of TAFAZZIN and NLRX1 together (*p* < 0.01 or *p* < 0.001 vs. hypoxia group). TRMT10C knockdown also suppressed the production of IL-1β and TNF-α under hypoxic conditions (*p* < 0.001 vs. hypoxia group). However, this effect was attenuated by TAFAZZIN knockdown (*p* < 0.05 vs. hypoxia + TRMT10C knockdown), NLRX1 knockdown (*p* < 0.05 or *p* < 0.01 vs. hypoxia + TRMT10C knockdown), as well as the knockdown of TAFAZZIN and NLRX1 together (*p* < 0.01 or *p* < 0.001 vs. hypoxia + TRMT10C knockdown). Hypoxia dramatically induced the production of ROS in AC16 and H9c2 cells, which was suppressed by the overexpression of TAFAZZIN and NLRX1 alone and in combination (*p* < 0.01, or *p* < 0.001 vs. hypoxia group). TRMT10C knockdown also lowered ROS levels in hypoxic cells (*p* < 0.001 vs. hypoxia group). This effect was attenuated by the knockdown of TAFAZZIN and NLRX1 alone (*p* < 0.01 vs. hypoxia + TRMT10C knockdown) or in combination (*p* < 0.001 vs. hypoxia + TRMT10C knockdown). Taken together, the reduction in TAFAZZIN and NLRX1 expression mediates the detrimental effects of TRMT10C in cardiomyocytes under hypoxia.

### TAFAZZIN and NLRX1 regulate autophagy and mitophagy in cardiomyocytes under hypoxia

**Figure 8:**
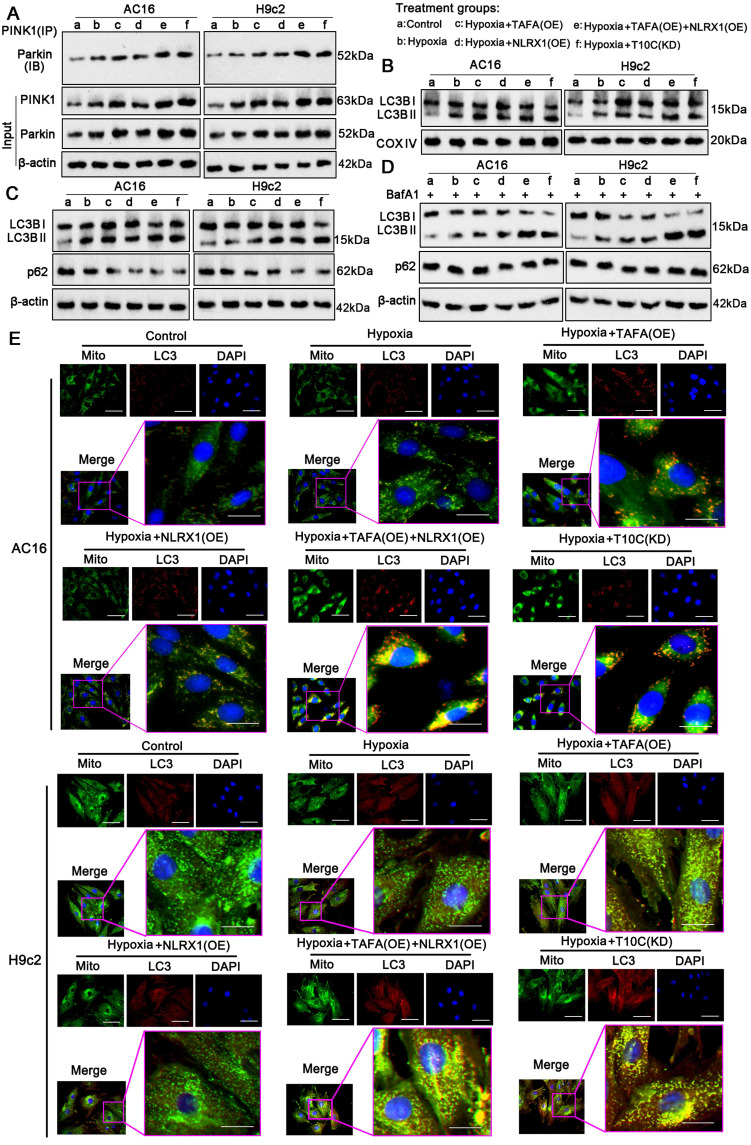
** TAFAZZIN and NLRX1 regulate mitophagy in cardiomyocytes under hypoxia.** AC16 and H9c2 cells were transfected with expression vectors for TAFAZZIN and NLRX1 to prevent their reduction under hypoxic conditions. Additionally, TRMT10C was knocked down in AC16 and H9c2 cells under these conditions. A. Co-immunoprecipitation (Co-IP) and western blot assays were performed to evaluate the interaction between PINK1 and Parkin, as well as their protein levels in AC16 and H9c2 cells. B. The protein levels of LC3B in the mitochondrial fraction were assessed by western blotting, with COV IV protein levels serving as the loading control. C. The overall protein levels of LC3B and p62 in the cells were detected by western blotting. D. A lysosomal inhibitor, bafilomycin A1 (BafA1, 20 nM), was applied to block the autophagy/mitophagy flux. Following this treatment, the overall protein levels of LC3B and p62 in the cells were analyzed by western blotting. E. Immunofluorescence using the MitoMark probe and anti-LC3 antibody was conducted to observe the interaction between mitochondria (green fluorescence) and autophagosomes (red fluorescence). The scale bar in the figures represents 10 μm. Abbreviations: T10C: TRMT10C; TAFA: TAFAZZIN; KD: Knockdown; OE: Overexpression.

Mitophagy is an important protective mechanism that prevents cells from undergoing apoptosis due to mitochondrial damage 27. TAFAZZIN and NLRX1 have been reported to regulate autophagy and mitophagy 28, 29. In this study, we aimed to determine the effects of TAFAZZIN and NLRX1 on mitophagy in cardiomyocytes under hypoxic conditions. PINK1-Parkin is an important initial signal of mitophagy. The protein levels of PINK1 and Parkin increased in AC16 and H9c2 cells under hypoxic conditions (*p* < 0.05, *p* < 0.01; Figure 8A and S9A). Overexpression of TAFAZZIN further increased PINK1 and Parkin protein levels under hypoxia (*p* < 0.01 or *p* < 0.001 vs. hypoxia), whereas overexpression of NLRX1 only increased Parkin in AC16 cells under hypoxia (*p* < 0.05). Overexpression of TAFAZZIN and NLRX1 also increased PINK1 and Parkin protein levels under hypoxic conditions (*p* < 0.01 or *p* < 0.001, respectively). In addition, TRMT10C knockdown induced an increase in PINK1 and Parkin protein levels under hypoxic conditions (*p* < 0.01 or *p* < 0.001). Co-IP assays indicated that TAFAZZIN overexpression alone or in combination with NLRX1 overexpression promoted the binding of PINK1 to Parkin under hypoxia. Similar results were observed after the TRMT10C knockdown. We further analyzed the accumulation of LC3B in mitochondria. As shown in Figure 8B and S9B, hypoxia-induced the accumulation of LC3B-II in the mitochondria (*p* < 0.05, *p* < 0.01). The accumulation of LC3B-II in the mitochondria was further increased by TAFAZZIN overexpression (*p* < 0.05, *p* < 0.01), NLRX1 overexpression (*p* < 0.05 in AC16 cells), combined overexpression of TAFAZZIN and NLRX1 (*p* < 0.01 or *p* < 0.001), and TRMT10C knockdown (*p* < 0.01, *p* < 0.001) under hypoxia. LC3B-Ⅱ and p62 protein levels are indicators of both autophagy and mitophagy because autophagy and mitophagy share the same steps for proteins and mitochondria to form autophagosomes and autolysosomes. The levels of LC3B Ⅱ and p62 in the total cell lysates were evaluated by western blotting (Figure 8C and Figure S9C). Under hypoxic conditions, LC3Ⅱ was increased in AC16 and H9c2 cells (*p* < 0.05), with a reduction in p62 (*p* < 0.05). Overexpression of TAFAZZIN increased LC3Ⅱ in AC16 cells (*p* < 0.05) and decreased p62 levels (*p* < 0.05) under hypoxia. Overexpression of NLRX1 increased LC3Ⅱ (*p* < 0.05) and decreased p62 (*p* < 0.05 or *p* < 0.01) in H9c2 cells. Co-overexpression of TAFAZZIN and NLRX1 and knockdown of TRMT10C increased LC3Ⅱ (*p* < 0.01, *p* < 0.001 vs. hypoxia) and decreased p62 expression (*p* < 0.01, or *p* < 0.001 vs. hypoxia) in both AC16 and H9c2 cells. LC3B II and p62 protein levels are influenced by lysosomal synthesis and degradation. As an autophagy inhibitor, BafA blocks autolysosomal degradation and inhibits autophagosome-lysosome fusion. In this study, treatment with BafA also resulted in an increase in LC3Ⅱ under hypoxia in AC16 and H9c2 cells (*p* < 0.01, Figure 8D and S9D). Furthermore, BafA treatment increased LC3Ⅱ levels caused by TAFAZZIN and NLRX1 overexpression and TRMT10C knockdown under hypoxia (*p* < 0.01 or *p* < 0.001 vs. hypoxia). However, the reduction in p62 levels caused by TAFAZZIN and NLRX1 overexpression and TRMT10C knockdown was suppressed or completely abolished by BafA treatment.

To further investigate mitophagy, we marked the mitochondria and autophagosomes with a MitoMark green probe and an anti-LC3 antibody, respectively, to observe their interaction by IF. The number of mitochondria (green fluorescence) colocalized with LC3 (red fluorescence) increased in AC16 and H9c2 cells under hypoxia (*p* < 0.05; Figure 8E). Co-localization was further increased by TAFAZZIN overexpression (*p* < 0.05, hypoxia), NLRX1 overexpression (*p* < 0.05, hypoxia), TAFAZZIN and NLRX1 co-overexpression (*p* < 0.01 or *p* < 0.001, hypoxia), and TRMT10C knockdown (*p* < 0.05, *p* < 0.01 vs. hypoxia). The intensity of yellow fluorescence, which merges green and red signals, was also enhanced by TAFAZZIN overexpression (*p* < 0.05, hypoxia), NLRX1 overexpression (*p* < 0.05 vs. hypoxia), TAFAZZIN and NLRX1 co-overexpression (*p* <0.01 or *p* < 0.001 vs. hypoxia), and TRMT10C knockdown (*p* < 0.05, *p* < 0.01 vs. hypoxia). The interaction between mitochondria and lysosomes was investigated using a MitoMark red probe and an anti-Lamp1 antibody via an IF. Lamp1 is a well-known lysosomal marker. As shown in Figure S10, hypoxia moderately promoted the overlap of red fluorescence (mitochondria) and green fluorescence (lysosomes), whereas TAFAZZIN and NLRX1 co-overexpression and TRMT10C knockdown strongly promoted this overlap. BafA treatment suppressed the overlap of red and green fluorescence induced by TAFAZZIN and NLRX1 overexpression and TRMT10C knockdown. Overall, TAFAZZIN and NLRX1 promoted autophagy and mitophagy in cardiomyocytes under hypoxic conditions. TRMT10C conversely suppressed autophagy and mitophagy under hypoxia, probably via the downregulation of TAFAZZIN and NLRX1.

### TAFAZZIN and NLRX1 regulate inflammatory signaling in the cardiomyocytes under hypoxia

**Figure 9:**
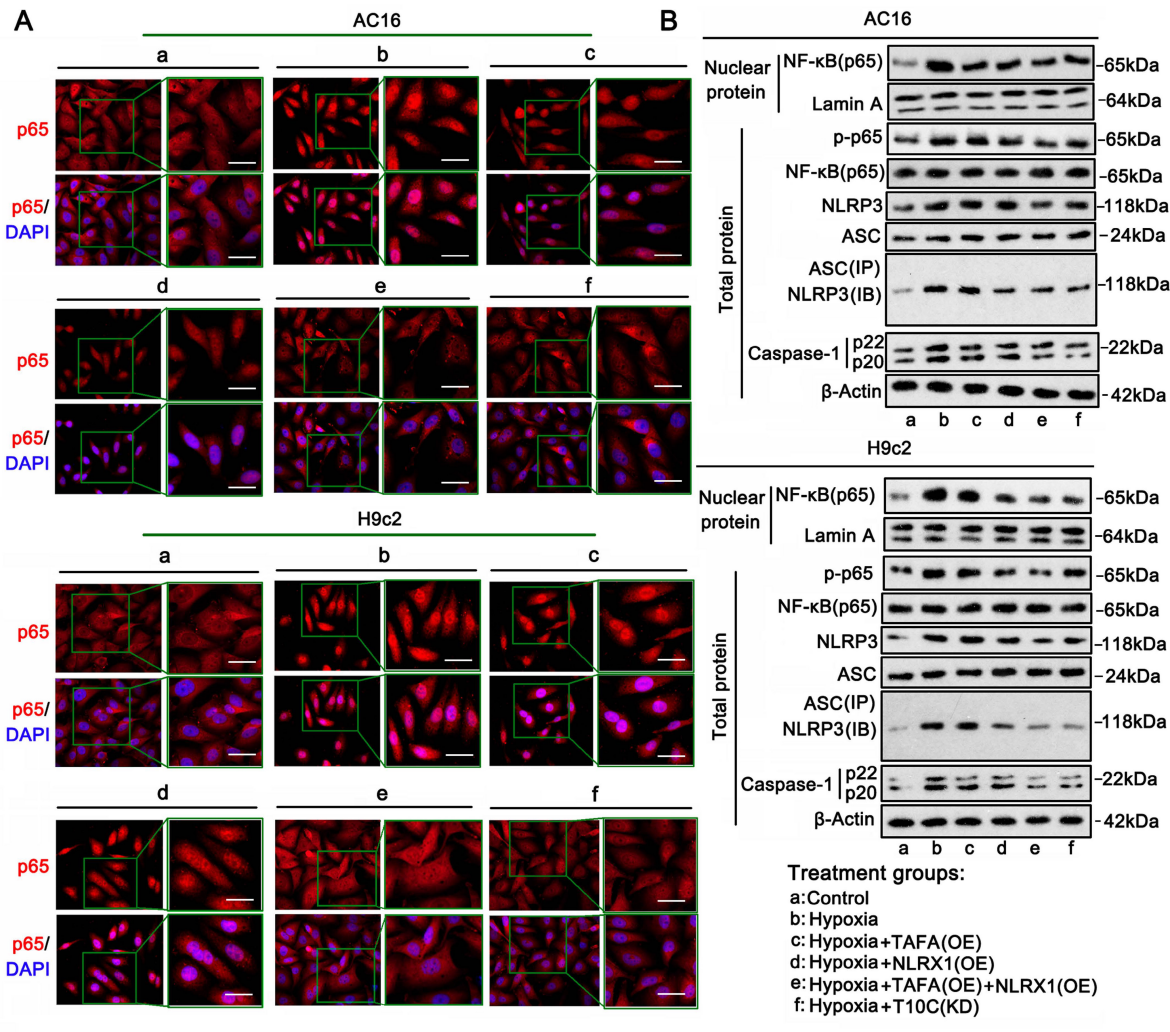
** TAFAZZIN and NLRX1 regulate inflammatory signaling in cardiomyocytes under hypoxia.** A. AC16 and H9c2 cells were transfected with expression vectors for TAFAZZIN and NLRX1 to prevent their reduction under hypoxic conditions. Additionally, TRMT10C was knocked down in AC16 and H9c2 cells under these conditions. Immunofluorescence analysis was performed to observe the distribution of p65 in the cytoplasm and nucleus. The scale bar in the figures represents 10 μm. B. Following similar cell treatments, western blotting was conducted to detect NF-κB (p65) in the cell nucleus, as well as p-p65, p65, NLRP3, ASC, and Caspase-1 in the cells. Co-immunoprecipitation (Co-IP) assays were performed to assess the interaction between NLRP3 and ASC.

NF-κB and NLRP3 signaling mediate most inflammatory responses in cells 20. Upon activation, NF-κB (p65) translocates from the cytoplasm to the nucleus, inducing the transcription of various inflammatory factors. IF analysis was performed to determine the distribution of p65 in the cytoplasm and nucleus. Hypoxia-induced accumulation of p65 in the nucleus was suppressed by TAFAZZIN overexpression, NLRX1 overexpression, TAFAZZIN and NLRX1 co-overexpression, and TRMT10C knockdown (Figure 9A). Western blotting was performed to quantitatively analyze the level of p65 in the cell nucleus (Figure 9B and S9E). Consistent with IF results, western blot analysis showed that hypoxia-induced increase of p65 in the nucleus was suppressed by TAFAZZIN overexpression (*p* < 0.01 vs. hypoxia only in AC16 cells), NLRX1 overexpression (*p* < 0.01 vs. hypoxia), TAFAZZIN and NLRX1 co-overexpression (*p* < 0.01 or *p* < 0.001 vs. hypoxia), and TRMT10C knockdown (*p* < 0.01 vs. hypoxia). Phosphorylation of p65 is an important mechanism underlying its transport to the nucleus. The level of phosphorylated p65 increased under hypoxic conditions, whereas the total level of p65 did not change. Increased phosphorylation of p65 was inhibited by TAFAZZIN overexpression (*p* < 0.01 vs. hypoxia only in AC16 cells), NLRX1 overexpression (*p* < 0.01 vs. hypoxia), TAFAZZIN and NLRX1 co-overexpression (*p* < 0.01, or *p* < 0.001 vs. hypoxia), and TRMT10C knockdown (*p* < 0.01 vs. hypoxia).

NLRP3 activation depends on binding to ASC. Activated NLRP3 stimulates caspase-1 to produce IL-1β and IL-18. Western blot analysis showed that NLRP3 expression increased in AC16 and H9c2 cells under hypoxia (*p* < 0.001). Co-overexpression of TAFAZZIN and NLRX1 decreased NLRP3 protein levels in AC16 and H9c2 cells (*p* < 0.001 vs. hypoxic conditions). In addition, TRMT10C knockdown decreased NLRP3 protein levels in AC16 and H9c2 cells (*p* < 0.01). ASC protein levels also increased in AC16 cells under hypoxia (*p* < 0.05) but not in H9c2 cells. Overexpression of TAFAZZIN and NLRX1 and knockdown of TRMT10C had no effect on ASC protein levels in AC16 and H9c2 cells. Co-immunoprecipitation analysis showed that the binding of NLRP3 to ASC increased in AC16 (*p* < 0.05) and H9c2 cells (*p* < 0.001 vs. hypoxia) under hypoxic conditions. TAFAZZIN overexpression did not suppress the binding of NLRP3 to ASC under hypoxic conditions. NLRX1 overexpression, alone or in combination with TAFAZZIN overexpression, suppressed the binding of NLRP3 to ASC under hypoxic conditions (*p* < 0.05, *p* < 0.01, respectively, vs. hypoxia). The binding of NLRP3 to ASC under hypoxic conditions was also inhibited by TRMT10C knockdown (*p* < 0.05, *p* < 0.01, respectively). Activated caspase-1 was increased in AC16 and H9c2 cells under hypoxic conditions (*p* < 0.001) and was suppressed by TAFAZZIN overexpression (*p* < 0.05), NLRX1 overexpression (*p* < 0.05), TAFAZZIN and NLRX1 co-overexpression (*p* < 0.01 or *p* < 0.001 vs. hypoxia), and TRMT10C knockdown (*p* < 0.01 vs. hypoxia). Collectively, TAFAZZIN and NLRX1 suppressed NF-κB-mediated inflammatory signals in cardiomyocytes under hypoxia. Similar effects were observed following the TRMT10C knockdown. NLRX1 overexpression also suppressed NLRP3-mediated inflammatory signals in cardiomyocytes under hypoxia, whereas TAFAZZIN overexpression probably had a weak effect on NLRP3 signals, as TAFAZZIN overexpression did not influence the protein levels of NLRP3 and ASC or their interaction.

### Knockout (KO) of TRMT10C attenuates heart damage in the CME model of mice

**Figure 10:**
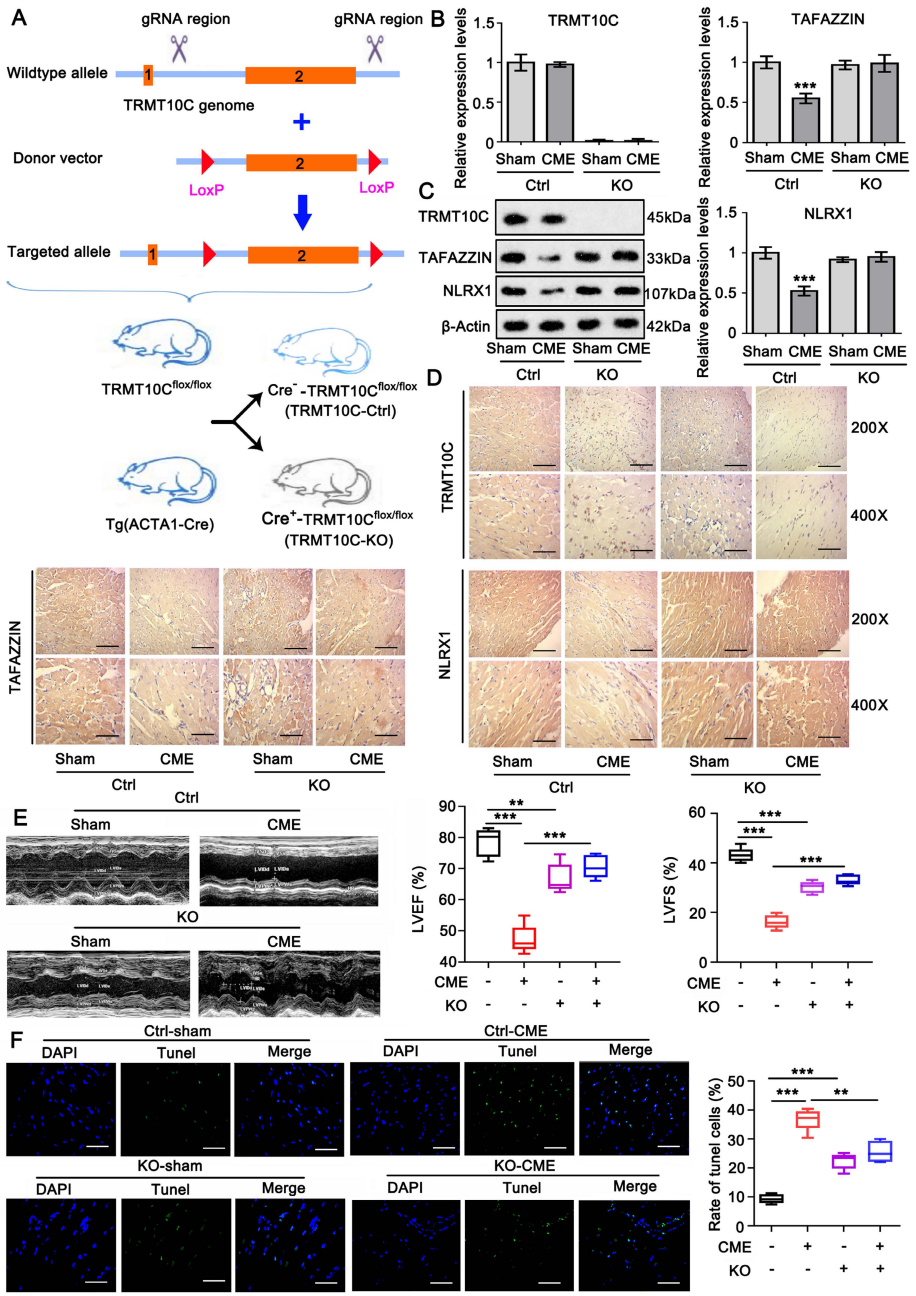
** Knockout (KO) of TRMT10C attenuates cardiac damage in a mouse model of CME.** A. The flowchart illustrates the process of TRMT10C knockout in C57BL/6 mice. B. A PCR assay was conducted to detect the mRNA levels of TRMT10C, TAFAZZIN, and NLRX1 in the sham and CME models of TRMT10C-Ctrl and TRMT10C-KO mice. C. Western blotting was performed to assess the protein levels of TRMT10C, TAFAZZIN, and NLRX1 in the sham and CME models of TRMT10C-Ctrl and TRMT10C-KO mice. D. Immunohistochemistry was conducted to detect TRMT10C, TAFAZZIN, and NLRX1 proteins in the sham and CME models of TRMT10C-Ctrl and TRMT10C-KO mice. The scale bar in the 200X images represents 120 μm, while the scale bar in the 400X images represents 50 μm. E. Echocardiography was used to measure the left ventricular ejection fraction (LVEF) and left ventricular fractional shortening (LVFS) to evaluate heart function. **p < 0.01 and ***p < 0.001 (n = 6). F. A TUNEL assay was conducted to assess apoptosis in heart tissues. The scale bar in the figures represents 50 μm. **p < 0.01 and ***p < 0.001 (n = 6).

The CRISPR/Cas9 system was used to establish TRMT10C-KO mice to verify the role of TRMT10C in CME. As shown in Figure 10A, LoxP sequences were inserted into the introns flanking exon 2 of TRMT10C to generate TRMT10C^flox/flox^ mice. TRMT10C^flox/flox^ mice were crossed with ACTA1-Cre mice to generate ACTA1-Cre^+^TRMT10C^flox/flox^ and ACTA1-Cre^-^-TRMT10C^flox/flox^ mice. Theoretically, TRMT10C is primarily lost in the heart tissues of ACTA1-Cre^+^-TRMT10C^flox/flox^ mice (TRMT10C-KO). ACTA1-Cre^-^-TRMT10C^flox/flox^ mice theoretically express TRMT10C in the heart tissue; therefore, these mice were used as controls (TRMT10C-Ctrl). We established CME models using TRMT10C-KO and TRMT10C-Ctrl mice. PCR detected the same levels of TRMT10C in sham and CME TRMT10C-Ctrl mice (Figure 10B). However, TRMT10C was undetectable in sham and CME TRMT10C-KO mice. TAFAZZIN and NLRX1 expression was reduced in the CME model of TRMT10C-Ctrl mice (*p* < 0.001) compared to sham TRMT10C-Ctrl mice. There was no significant difference in TAFAZZIN and NLRX1 expression between the sham and CME models in TRMT10C-KO mice. Similar to the RCR results, western blotting showed the same TRMT10C protein levels in the sham and CME models of TRMT10C-Ctrl mice (Figure 10C). TRMT10C was undetectable in sham and CME TRMT10C-KO mice. TAFAZZIN and NLRX1 protein levels were reduced in the CME model of TRMT10C-Ctrl mice compared to sham TRMT10C-Ctrl mice. There was no significant difference in TAFAZZIN and NLRX1 expression between the sham and CME models in TRMT10C-KO mice. As indicated by IHC, TRMT10C accumulated in the cytoplasm of sham TRMT10C-Ctrl mice, whereas TRMT10C accumulated in the nucleus of the CME model of TRMT10C-Ctrl mice (Figure 10D). TRMT10C-KO mice showed no or very weak expression of TRMT10C in heart tissues in the sham or CME models. TAFAZZIN and NLRX1 proteins were reduced in the CME model of TRMT10C-Ctrl mice but were not changed in the CME model of TRMT10C-KO mice.

Echocardiography was used to measure LVEF and LVFS to evaluate heart function. In TRMT10C-Ctrl mice, both LVEF and LVFS were reduced in the CME model compared to those in the sham group (*p* < 0.001; Figure 10E). LVEF and LVFS were also reduced in the sham group of TRMT10C-KO mice compared to those in the sham group of TRMT10C-Ctrl mice (*p* < 0.01 or *p* < 0.001). However, LVEF and LVFS levels in the CME model of TRMT10C-KO mice were much higher than those in the CME model of TRMT10C-Ctrl mice (*p* < 0.001). These results suggested that TRMT10C depletion improves heart function in patients with CME. TUNEL assay was conducted to evaluate apoptosis in heart tissues. In TRMT10C-Ctrl mice, apoptosis was much higher in the CME model group than in the sham group (*p* < 0.001; Figure 10F). TRMT10C KO also increased apoptosis compared to the sham group (*p* < 0.001 vs. sham group in TRMT10C-Ctrl mice). However, TRMT10C KO mice showed suppressed apoptosis in the CME model (*p* < 0.001 vs. the CME model in TRMT10C-Ctrl mice).

## Discussion

This study found reduced TRMT10C levels in the mitochondria but increased TRMT10C enrichment in the nucleus of cardiomyocytes in CME models both *in vivo* and *in vitro.* TRMT10C is a mitochondrial N(1)-methyltransferase that modifies both tRNAs and rRNAs. A later study found that TRMT10C could also induce m1A methylation of mRNA 30. The short mitochondrial genome (16.6 kb) encodes 22 tRNAs, 2 rRNAs, and 13 mRNAs, which together produce the essential components for oxidative phosphorylation. m1A modification is implicated in regulating the correct folding and structural stability of tRNAs. Metodiev et al. reported that TRMT10C mutations suppressed m1A modification of mitochondrial tRNAs, leading to multiple respiratory chain deficiencies, impaired oxidative phosphorylation, and insufficient ATP production 31. Reduced TRMT10C levels have also been observed in C57BL/6J 5XFAD mice, an animal model of Alzheimer's disease 32. A decrease in TRMT10C was associated with reduced levels of mature mitochondrial tRNA levels and several Alzheimer's-related phenotypes 32. However, the function of TRMT10C in the nucleus has rarely been reported, likely because TRMT10C is seldom localized to the nucleus under normal conditions. Moreover, the total TRMT10C level in cardiomyocytes from CME models did not differ from that in controls. Therefore, the increase in nuclear TRMT10C is likely associated with a corresponding decrease reduction in mitochondrial TRMT10C.

Except for a few proteins that are produced directly in the mitochondria, most proteins are synthesized in the cytoplasm via organelles such as ribosomes, the endoplasmic reticulum, and the Golgi apparatus. TRMT10C is encoded by nuclear DNA, not mitochondrial DNA. Therefore, like most proteins encoded by nuclear DNA, TRMT10C is produced in the cytoplasm rather than in the mitochondria. This study found that, in CME models, TRMT10C is transported from the cytoplasm to the nucleus rather than to the mitochondria. The underlying mechanism involves the suppression of TRMT10C protein succinylation, which promotes the interaction between the nuclear import receptor KPNA4 and TRMT10C.

Protein succinylation is affected by ischemic and hypoxic conditions. Chen et al. found that exposing N2a neuroblastoma cells to hypoxia for only 1 h enhanced protein succinylation 33. However, chronic myocardial ischemia suppresses protein succinylation, as validated in clinical samples from patients with ischemia-induced heart failure 7. Takada et al. observed a considerable reduction in myocardial succinyl-CoA levels following long-term coronary infarction in C57BL/6J mice 3.

Succinyl-CoA is an essential substrate for succinyl transferases, which transfer succinyl groups to lysine residues during protein succinylation 34. In this study, we found that succinyl-CoA levels increased after short-term exposure of cardiomyocytes to hypoxia. This increase may reflect a stress response of cardiomyocytes to hypoxia; however, the precise mechanisms require further investigation. The TCA cycle, which is the primary source of succinyl CoA, is an oxygen-dependent process. Prolonged hypoxia undoubtedly inhibits the TCA cycle. Notably, succinyl-CoA was considerably reduced after 48 h of hypoxic culture. The downregulation of CPT1A likely contributed to the reduction in succinyl-CoA, which in turn suppressed protein succinylation—observed both in the animal CME model and in cardiomyocytes exposed to prolonged hypoxia. This reduction in CPT1A likely led to decreased succinylation of TRMT10C protein, as overexpression of CPT1A partially restored TRMT10C succinylation in hypoxic cardiomyocytes. Promoting TRMT10C protein succinylation increased its accumulation in the mitochondria and decreased its presence in the nucleus under hypoxic conditions. These findings suggest that protein succinylation influences the intracellular distribution of TRMT10C between the mitochondria and the nucleus.

This study showed that the CPT1A-induced succinylation of TRMT10C suppressed the binding of KPNA4 to TRMT10C. Succinylation refers to the addition of succinyl groups to lysine residues. Protein lysine residues generally have a positive charge, but the positive charge can be neutralized by PTMs such as succinylation, acetylation, and methylation, leading to a shift in protein structure and enzymatic properties. Among these PTMs, the succinate group is the largest, with approximately 100.02 Daltons (Da) 4. For other typical covalent modification groups of lysine, acetyl is 42.0106 Da, and dimethyl is 28.0313 Da 4. Succinyl-CoA is the most abundant acyl-CoA in the heart 35. Therefore, succinylation may exert a larger force and affect the structure and function of target proteins compared to other PTMs. The KPNA family of proteins, which bind to cargo proteins, rely on the recognition of NLS in the cargo protein. We identified two potential NLS in human TRMT10C. One of the NLS (KAKR) is conserved and exists in mouse and rat TRMT10C proteins. The succinylation modification site (K325-Succ2) is located within the NLS (K325AKR). Mutations at this succinylation site attenuate the binding of KPNA4 to both human and rat TRMT10C proteins, even when CPT1A is overexpressed. These results suggest that the succinylation of K325 blocks the binding of KPNA4 to TRMT10C. This effect is likely associated with the succinyl group added to K325, which prevents KPNA4 from recognizing NLS (KAKR). Alternatively, the protein conformation may change after succinylation, influencing KPNA4 binding to TRMT10C. Another potential NLS in the human TRMT10C protein [KKK(X)_10_KVKK, where X is any other amino acid] is close to another succinylation modification site (K173-Succ1). However, K173-Succ1 does not exist in mouse and rat TRMT10C proteins. We found that the mutation at K173 also attenuated the binding of KPNA4 to the human TRMT10C protein, even though CPT1A was overexpressed. Succinylation of both K173 and K325 prevents the binding of KPNA4 to the human TRMT10C protein, leading to a reduction in the human TRMT10C protein levels in the cell nucleus. As there is one more succinylation modification site in the human TRMT10C protein, sensitivity to the reduction in CPT1A-mediated succinylation may differ in AC16 and H9c2 cells. With succinylation, the amount of TRMT10C protein increased in the mitochondria but not in the cytoplasm. Therefore, when TRMT10C is not transported to the nucleus, TRMT10C is more likely to be transported to the mitochondria rather than remaining in the cytoplasm. It remains unclear how TRMT10C is transported from the cytoplasm to the mitochondria.

The altered distribution of TRMT10C between the mitochondria and nucleus likely plays an important role in the pathogenesis of CME. As the m1A modification of tRNAs is important for their role in energy metabolism 36, the deficiency of TRMT10C in mitochondria likely exerts detrimental effects on cardiomyocytes. Under normoxia, transfection with siRNA-TRMT10C caused a substantial reduction in mitochondrial TRMT10C expression. Consequently, cardiomyocyte viability and proliferation were suppressed, the apoptosis rate increased, and the production of inflammatory factors and ROS in cells was elevated. To determine the effect of increased TRMT10C levels in the nucleus, cardiomyocytes were cultured under hypoxic conditions. Transfection with siRNA-TRMT10C suppressed the hypoxia-induced accumulation of TRMT10C in the nucleus. Moreover, TRMT10C knockdown improved cardiomyocyte viability and proliferation under hypoxia and suppressed apoptosis compared to the hypoxia group. These results suggest that an increase in nuclear TRMT10C expression is detrimental to cardiomyocytes. Overexpression of CPT1A can restore TRMT10C succinylation under hypoxic conditions, thereby restoring TRMT10C levels in the mitochondria and reducing its level in the nucleus. Therefore, CPT1A overexpression improved the status of cardiomyocytes under hypoxia, though this effect was abrogated by TRMT10C knockdown. CPT1A also plays an important role in regulating mitochondrial fatty acid oxidation; however, the oxidation of fatty acids and subsequent oxidative phosphorylation require oxygen. Therefore, under hypoxic conditions, the protective effects of CPT1A may depend on its succinyl transferase function.

This study further investigated why an increase in nuclear TRMT10C was detrimental to cardiomyocytes. Compared to the nucleus, the substrates of TRMT10C (tRNA and mRNA) are limited in the mitochondria. When TRMT10C accumulates in the cell nucleus, it can regulate more RNAs there than in the mitochondrion by acting as an 'm1A writer.' In this study, TAFAZZIN and NLRX1 mRNAs were identified as targets of TRMT10C in the nucleus. Neither TAFAZZIN nor NLRX1 mRNA was detected in the mitochondria. Some reports have shown that m1A can directly disrupt the formation of Watson-Crick base pairs, leading to strand elongation and nucleotide misincorporation 37. Other studies have shown that m1A provides recognition sites for 'm1A readers' in mRNAs 38, 39. These m1A readers further determine the characteristics of the mRNA, such as its stability and translation. For example, m1A modification impairs E2F1 mRNA stability and suppresses ATP5D mRNA translation by recruiting the m1A reader YTHDF1, which facilitates ATP5D mRNA release from the ribosome complex 38. The m1A demethylase AlkB homolog 1 promotes the expression of colony-stimulating factor 1 and methyltransferase-like 3, likely by increasing their mRNA stability 39, 40. In our study, we found that TRMT10C-mediated m1A modification promoted the binding of YTHDF2 to TAFAZZIN and NLRX1 mRNAs. YTHDF2 induces mRNA degradation, thereby suppressing its expression. YTHDF2, which induces decay of m1A-modified mRNA, was also identified in a study by Seo and Kleiner 41. YTHDF2 knockdown increases m1A-modified mRNA expression. In addition to YTHDF2, the m1A reader YTHDF3 has also been reported to destabilize IGF1R mRNA, resulting in the downregulation of IGF1R in trophoblasts 42. In our study, the TRMT10C-mediated m1A expression of TAFAZZ mRNA was also recognized by YTHDF3. However, the knockdown of YTHDF3 alone was insufficient to reverse TAFAZZ mRNA expression under hypoxic conditions.

Notably, YTHDF2 functions as both an m6A and m1A reader 43. Using the m6A dot blot assay, we noticed that the overall m6A level in mRNA increased in cardiomyocytes after exposure to hypoxia. It appears that the m6A modification may also contribute to the suppressive effect of YTHDF2 on TAFAZZIN and NLRX1. However, after the knockdown of TRMT10C, overexpression of YTHDF2 had no effect on the expression of TAFAZZIN or NLRX1. In addition, TAFAZZIN and NLRX1 were not regulated by TRMT10C or YTHDF2 after the m1A sites on the mRNAs were mutated. These results suggest that the suppressive effect of YTHDF2 on TAFAZZIN and NLRX1 is solely dependent on m1A modifications. To understand the role of m1A modifications in mRNA translation, we conducted a polysome profiling assay. These results showed that the TRMT10C-mediated m1A modification had no effect on the translation of TAFAZZIN and NLRX1.

TAFAZZIN is an important mitochondrial enzyme responsible for transferring fatty acids from phospholipids to lysophospholipids. The TAFAZZIN mutation has been associated with Barth Syndrome 44, a genetic disorder where patients often develop dilated cardiomyopathy and die from cardiac decompensation during infancy or early childhood. Pathological studies have shown that patients lack mature cardiolipin (CL), which is abundant in cardiac muscles 44, 45. The synthesis of mature CL relies on TAFAZZIN, which converts monolyso-cardiolipin (a CL derivative) to mature CL 46. Mature CL is a key component of the mitochondrial membrane and is essential for the proper structure and function of the electron transport chain assembly, which synthesizes ATP. Abnormalities in CL levels, structure, or localization in the mitochondria can lead to mitochondrial dysfunction, excessive ROS production, release of cytochrome C for apoptosis, and abnormal mitophagy 47. In the present study, TAFAZZIN expression was reduced in cardiomyocytes exposed to hypoxia. TAFAZZIN overexpression suppressed ROS production in cardiomyocytes and limited apoptosis. Mitophagy was enhanced by TAFAZZIN overexpression. A study suggests that impaired mitochondria release intramitochondrial CL to the outer surface of mitochondria, where CL contributes to the recognition of damaged mitochondria and the initiation of autophagosome formation, ultimately leading to mitophagy 47. Immediate removal of damaged mitochondria is vital for cell survival because it prevents the release of ROS and cytochrome C, which induce various detrimental signals.

NOD-like receptors (NLRs) are intracellular sensors that recognize a variety of pathogen-associated molecular patterns and exogenous/endogenous damage-associated molecular patterns, playing an important role in regulating innate immune responses. As an NLR, NLRX1 is the only member primarily localized to the mitochondria and has diverse cellular functions, including anti-inflammatory effects, regulation of energy metabolism and ROS production, and induction of autophagy 48-50. In this study, we observed reduced NLRX1 expression in cardiomyocytes under hypoxic conditions. However, overexpression of NLRX1 suppressed apoptosis, ROS production, and inflammation while enhancing autophagy and mitophagy. Overexpression of both NLRX1 and TAFAZZIN exerted considerable beneficial effects. TRMT10C knockdown also increased NLRX1 and TAFAZZIN expression and showed improved effects, whereas the knockdown of NLRX1 and TAFAZZIN abolished the beneficial effects of TRMT10C knockdown. These results suggest that the increase in NLRX1 and TAFAZZIN mediated the improvement observed with TRMT10C knockdown. Conversely, the accumulation of TRMT10C in the nucleus induces cardiomyocyte damage under hypoxia by reducing NLRX1 and TAFAZZIN expression.

TRMT10C knockdown suppressed the accumulation of TRMT10C in the nucleus. It restored NLRX1 and TAFAZZIN protein levels in cardiomyocytes under hypoxia. However, the deficiency of TRMT10C in the mitochondria did not improve or even worsened with TRMT10C knockdown. Therefore, inducing TRMT10C succinylation via CPT1A overexpression may be a better approach for improving cardiomyocytes under hypoxia than direct knockdown of TRMT10C.

This study had some limitations worth noting. Although it elucidated the mechanism by which TRMT10C is transported to the nucleus by KPNA4 after the suppression of TRMT10C succinylation, the reason why TRMT10C is lost in the mitochondria remains unclear. Two possible mechanisms could explain this phenomenon. First, CPT1A is primarily located in the mitochondria but also in the cytoplasm, where it succinylates proteins not located in the mitochondria, such as ATG16L1 and S100A10 51, 52. It is possible that reduced CPT1A levels in the cytoplasm led to decreased succinylation of newly produced TRMT10C, causing its translocation to the nucleus instead of the mitochondria. Another possibility is that TRMT10C is translocated and maintained in the mitochondria. A reduction in TRMT10C succinylation due to reduced CPT1A in the mitochondria may cause the release of TRMT10C from the mitochondria into the cytoplasm, followed by its transport to the nucleus. All these possibilities will be explored in future studies. In this study, TRMT10C KO mice were created using ACTA1-Cre mice. The *ACTA1* promoter is active in both the heart and skeletal muscles, which may have influenced the skeletal muscle phenotype and, ultimately, the cardiac phenotype in TRMT10C KO mice.

**Figure 11:**
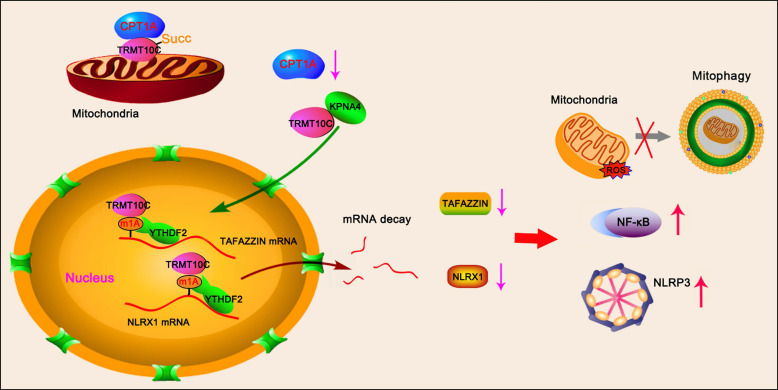
** Mechanistic diagram illustrating the detrimental effects of TRMT10C in the nucleus under hypoxic conditions via m1A modification.** CPT1A is a succinyltransferase responsible for protein succinylation. The reduction in CPT1A and succinyl-CoA levels inhibits the succinylation of TRMT10C in cardiomyocytes during CME. This suppression of TRMT10C succinylation enhances its binding to KPNA4, facilitating the transport of TRMT10C from the cytoplasm to the nucleus instead of to the mitochondria. In the nucleus, TRMT10C promotes the decay of TAFAZZIN and NLRX1 through m1A modification. The reduction of TAFAZZIN and NLRX1 is associated with impaired mitophagy, which may lead to increased apoptosis and reactive oxygen species (ROS) production. Additionally, the decrease in TAFAZZIN and NLRX1 facilitates the activation of inflammatory signaling pathways, such as NF-κB and NLRP3. The reduced levels of NLRX1 promote the interaction between ASC and NLRP3, thereby activating the NLRP3 signaling pathway and inducing the production of inflammatory factors.

Overall, the reduction in CPT1A and succinyl-CoA levels suppressed TRMT10C succinylation in CME cardiomyocytes. Suppression of succinylation in TRMT10C promotes its binding to KPNA4, facilitating the transport of TRMT10C from the cytoplasm to the nucleus instead of the mitochondria. TRMT10C in the nucleus causes the decay of TAFAZZIN and NLRX1 through m1A modification. The reduction in TAFAZZIN and NLRX1 is associated with multiple detrimental effects, such as inflammation mediated by NF-κB and NLRP3, ROS production, and suppression of mitophagy. A diagram of this mechanism is shown in Figure 11. This study reveals a novel pathological mechanism underlying CME and suggests therapeutic targets for this disease.

## Supplementary Material

Supplementary figures and tables.
