# Supplementary material for: De-succinylation-induced accumulation of TRMT10C in the nucleus plays a detrimental role in coronary microembolization via its m1A modification function
Source: Int J Biol Sci. 2025 Apr 13;21(7):2891–920. doi: 10.7150/ijbs.107965 (PMC12080399; doi:10.7150/ijbs.107965)
Supplement: Supplementary file 1 — Supplementary figures and tables. [file ijbsv21p2891s1.pdf]

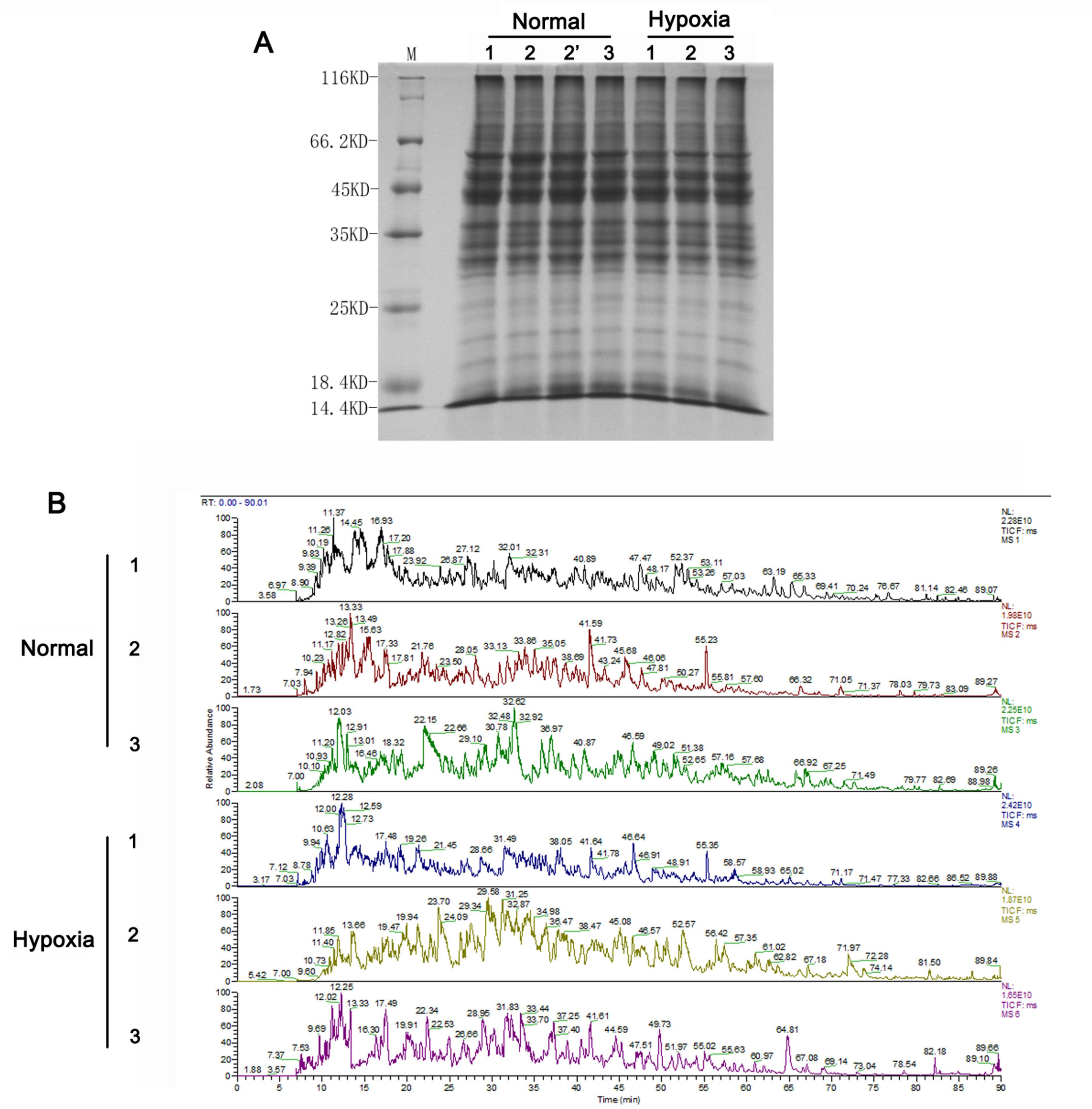

Supplementary Figure 1. Proteomic assay in AC16 cells under normal and hypoxia conditions

A, AC16 cells cultured under normal and hypoxia conditions. Three or four cell samples from each condition were collected for the proteomic assay. Polyacrylamide gel electrophoresis is conducted to separate proteins with different molecular weight.

B, Protein mass spectrometry analysis.

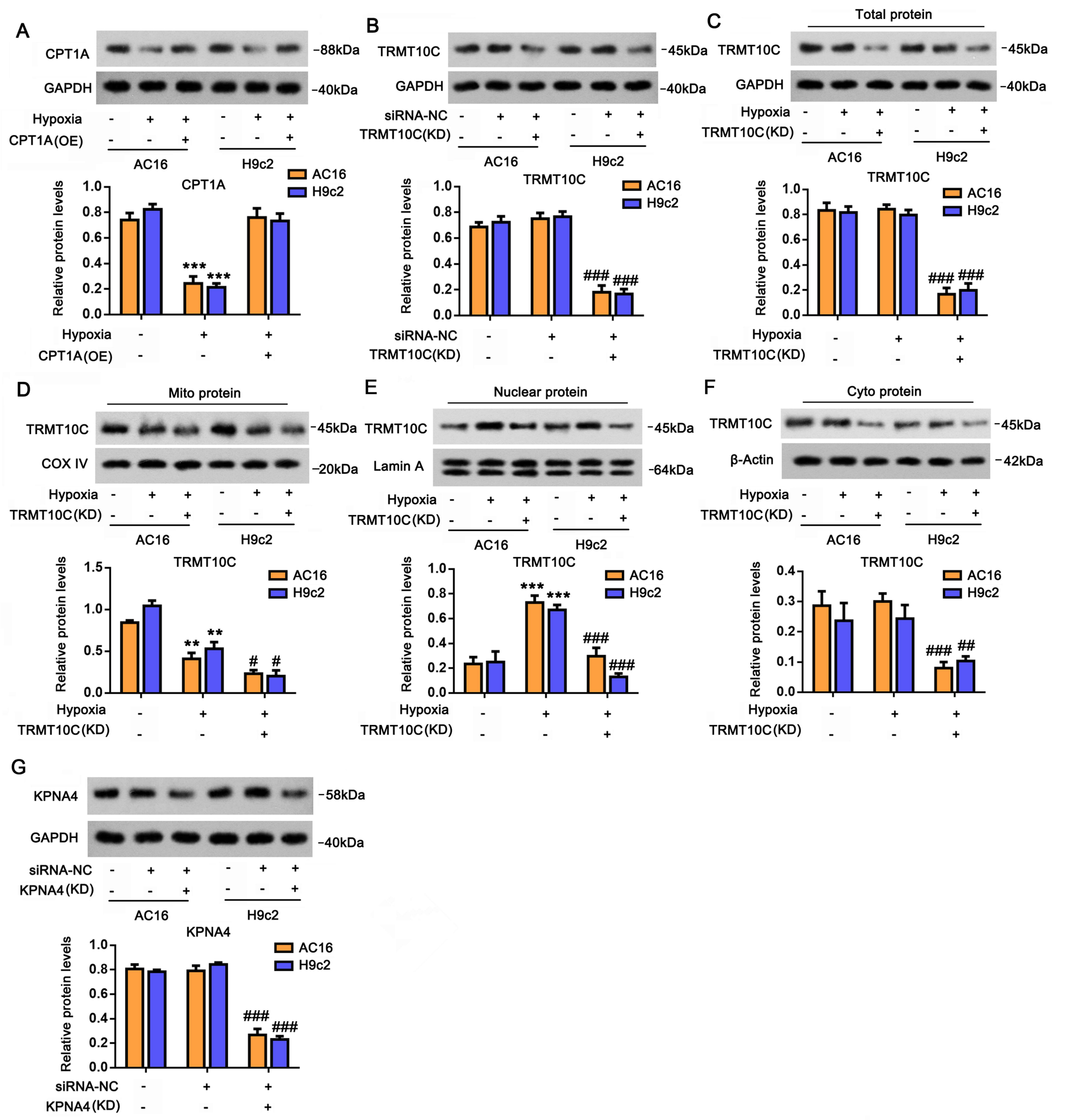

Supplementary Figure 2. Western blot detection of proteins after indicated genes were knocked down or overexpressed.

A, Western blot detection of CPT1A after the overexpression in AC16 and H9c2 cells under the hypoxic condition. ***$p < 0.001$ vs. control (n = 3).

B, Western blot detection of TRMT10C after the cell transfection with siRNA-negative-control (NC) and siRNA-targeting TRMT10C. ###$p < 0.001$ vs. control (n = 3).

C-F, TRMT10C was knocked down in AC16 and H9c2 cells under the hypoxic condition. Western blot detection of the total TRMT10C in cells and it in cell fractions including mitochondria, nucleus and cytoplasm (without mitochondria). **$p < 0.01$,

****p* < 0.001 vs. control group (n = 3); #*p* < 0.05, ##*p* < 0.01, ###*p* < 0.001 vs. Hypoxia group (n = 3).

G, Western blot detection of KPNA4 after the cell transfection with siRNA-NC and siRNA-targeting KPNA4.###*p* < 0.001 vs. control (n = 3).

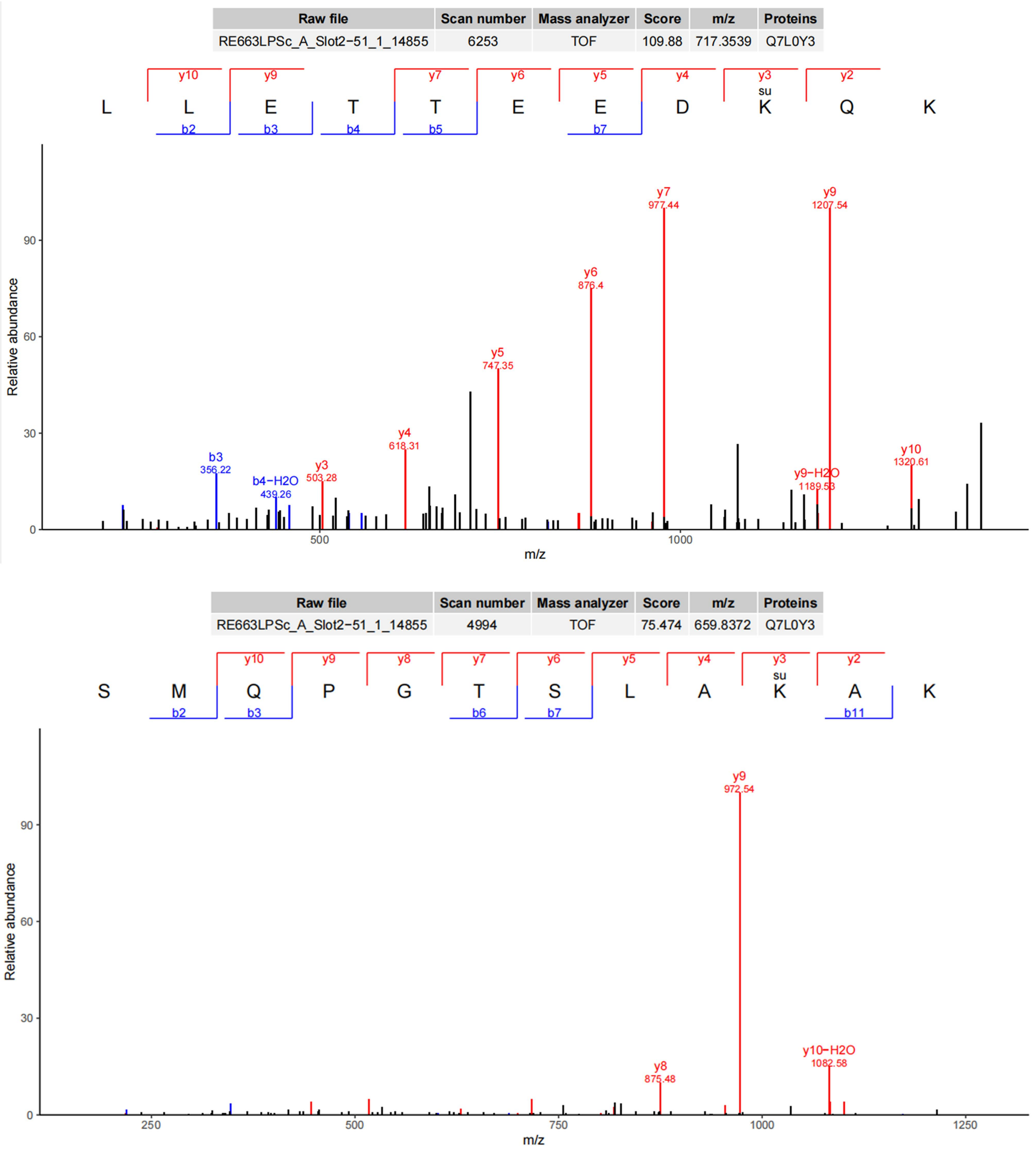

Supplementary Figure 3. Mass spectrometric analysis of succinylation location in the human TRMT10C protein

Mass spectrometric analysis showed succinylation at K173 and K325 in the human TRMT10C protein.

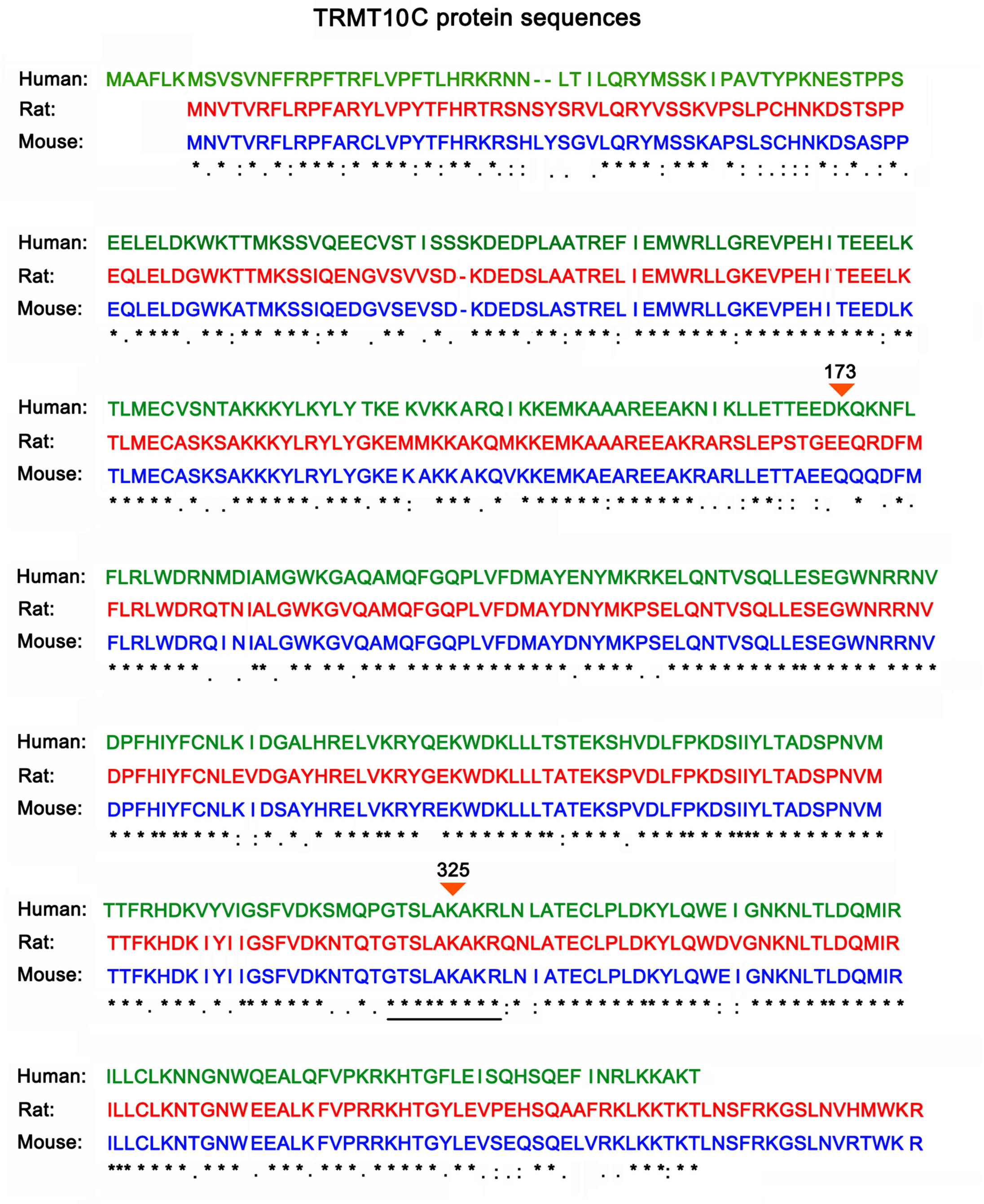

Supplementary Figure 4. The amino acid sequences of TRMT10C proteins in human, rat and mouse species.

TRMT10C lacks K173 in rat and mouse species. However, K325 and the adjacent amino acid sequence (GTSLAK325AKR) are highly conserved among the three species.

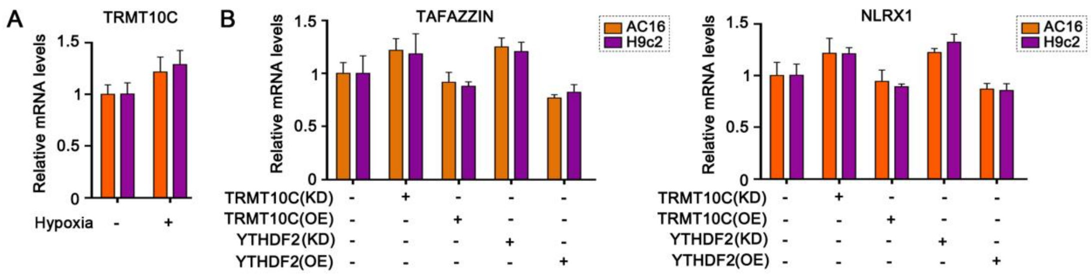

Supplementary Figure 5. The expression of TAFAZZIN and NLRX1 after the knockdown and overexpression of TRMT10C and YTHDF2 in cells under the normoxic condition

A, PCR was performed to detect the mRNA levels of TRMT10C under the hypoxic normoxic conditions.

B, PCR was performed to detect the expression of TAFAZZIN and NLRX1 after the knockdown and overexpression of TRMT10C and YTHDF2 in cells under the normoxic condition.

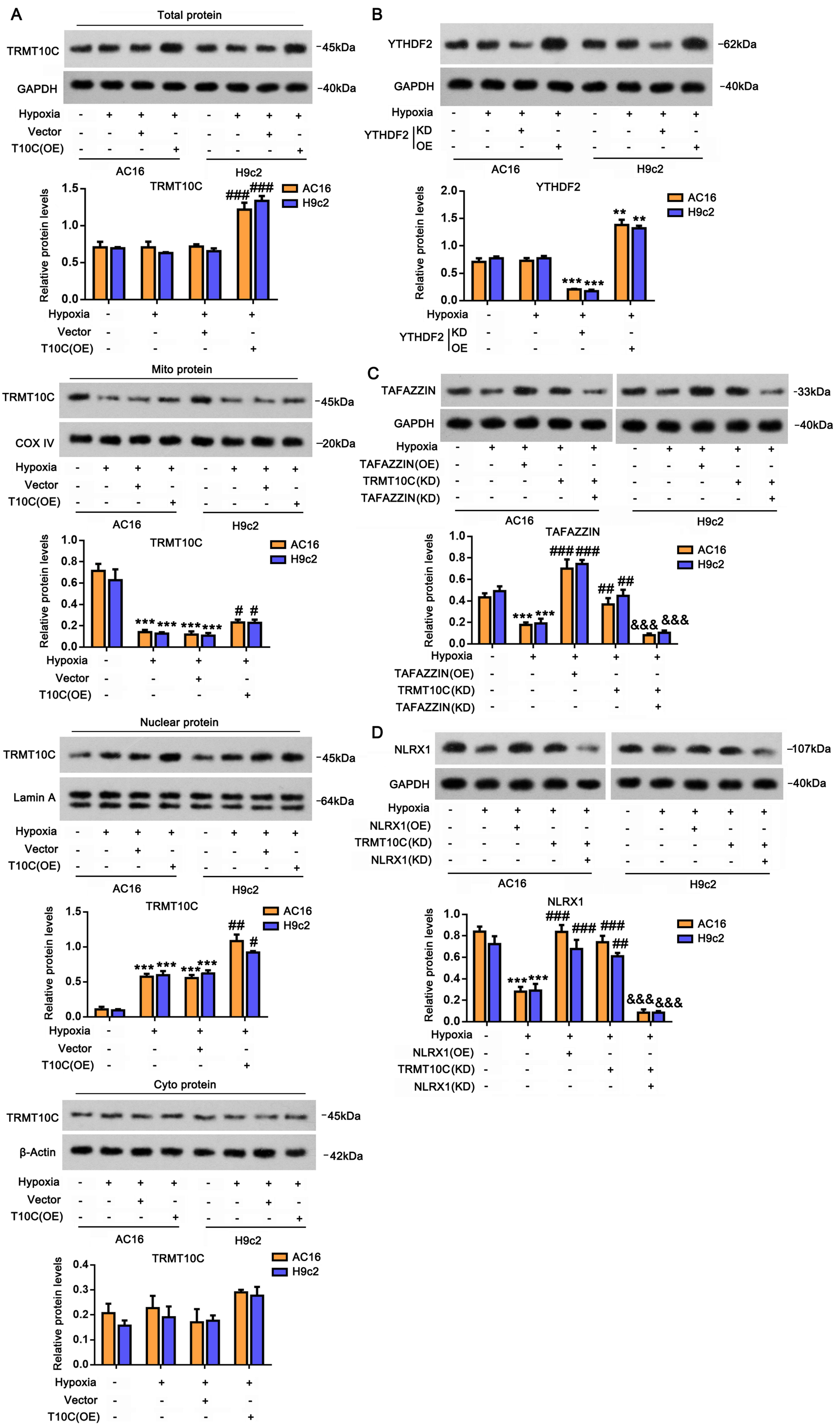

A
Total protein
TRMT10C
GAPDH
-45kDa
-40kDa
Hypoxia
Vector
T10C(OE)
AC16
H9c2
TRMT10C
AC16
H9c2
Relative protein levels
###
###
Mito protein
TRMT10C
COX IV
-45kDa
-20kDa
***
#
Nuclear protein
Lamin A
-64kDa
##
Cyto protein
β-Actin
-42kDa
B
YTHDF2
-62kDa
YTHDF2 KD
OE
**
C
TAFAZZIN
-33kDa
TAFAZZIN(OE)
TRMT10C(KD)
TAFAZZIN(KD)
&&&
D
NLRX1
-107kDa
NLRX1(OE)
NLRX1(KD)

Supplementary Figure 6. Western blot detection of proteins after indicated genes were knocked down or overexpressed under the hypoxic condition

A, TRMT10C was overexpressed in AC16 and H9c2 cells under the hypoxic condition. Western blot detection of the total TRMT10C in cells and it in cell fractions including mitochondria, nucleus and cytoplasm (without mitochondria). ***$p < 0.001$ vs. control group (n = 3); #$p < 0.05$, ##$p < 0.01$, ###$p < 0.001$ vs. Hypoxia group (n = 3).

B, Western blot detected YTHDF2 after it was knocked down and overexpressed in AC16 and H9c2 cells under the hypoxic condition. **$p < 0.01$, ***$p < 0.001$ vs. control group (n = 3).

C, Western blot detected TAFAZZIN after it was overexpressed and knocked down with TRMT10C knockdown. ***$p < 0.001$ vs. control group; ##$p < 0.01$, ###$p < 0.001$ vs. Hypoxia group; &&&$p < 0.001$ vs. Hypoxia + TRMT10C knockdown group (n = 3).

D, Western blot detected NLRX1 after it was overexpressed and knocked down with TRMT10C knockdown. ***$p < 0.001$ vs. control group; ##$p < 0.01$, ###$p < 0.001$ vs. Hypoxia group; &&&$p < 0.001$ vs. Hypoxia + TRMT10C knockdown group (n = 3).

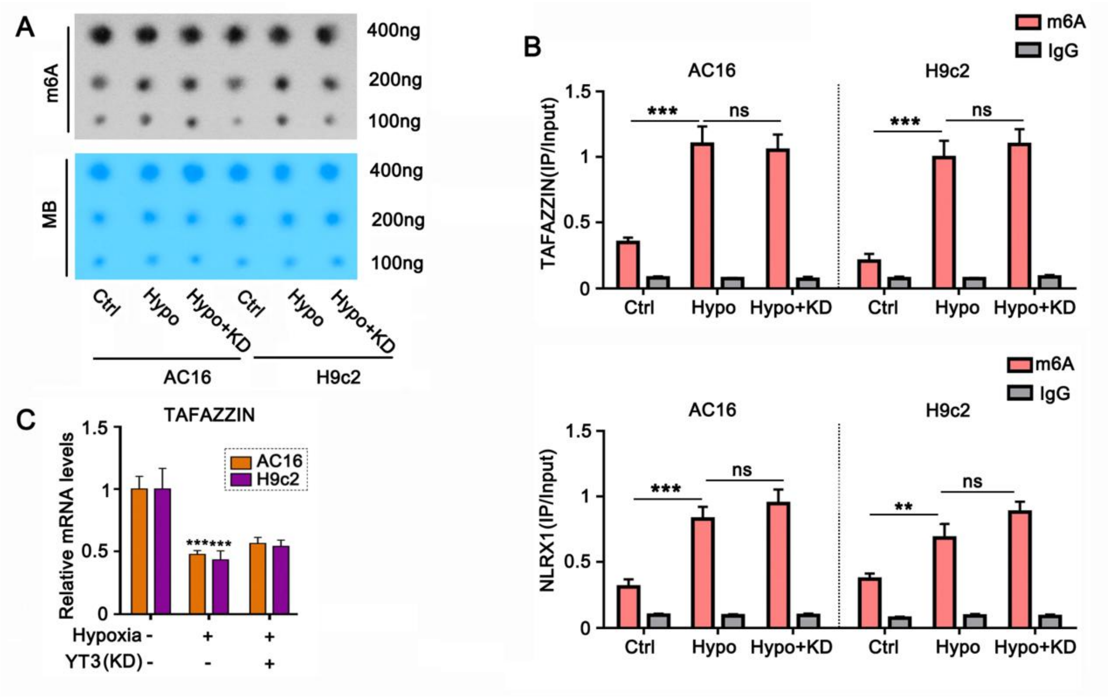

Supplementary Figure 7. The effect of m6A modification on the TAFAZZIN and NLRX1

A, TRMT10C was knocked down in AC16 and H9c2 cells under hypoxia. The whole m6A level was detected by m6A dot blot assay. The gray value of the dot blot was used to evaluate the m6A level. The same membrane was stained with 0.02% methylene blue (MB) as a loading control.

B, TRMT10C was knocked down in AC16 and H9c2 cells under hypoxia. RIP was conducted to determine the change of m6A level in TAFAZZIN and NLRX1 mRNAs. **$p < 0.01$ and ***$p < 0.001$ (n = 3).

C, YTHDF3 was knocked down in AC16 and H9c2 cells under hypoxia. PCR was performed to detect TAFAZZIN mRNA level.

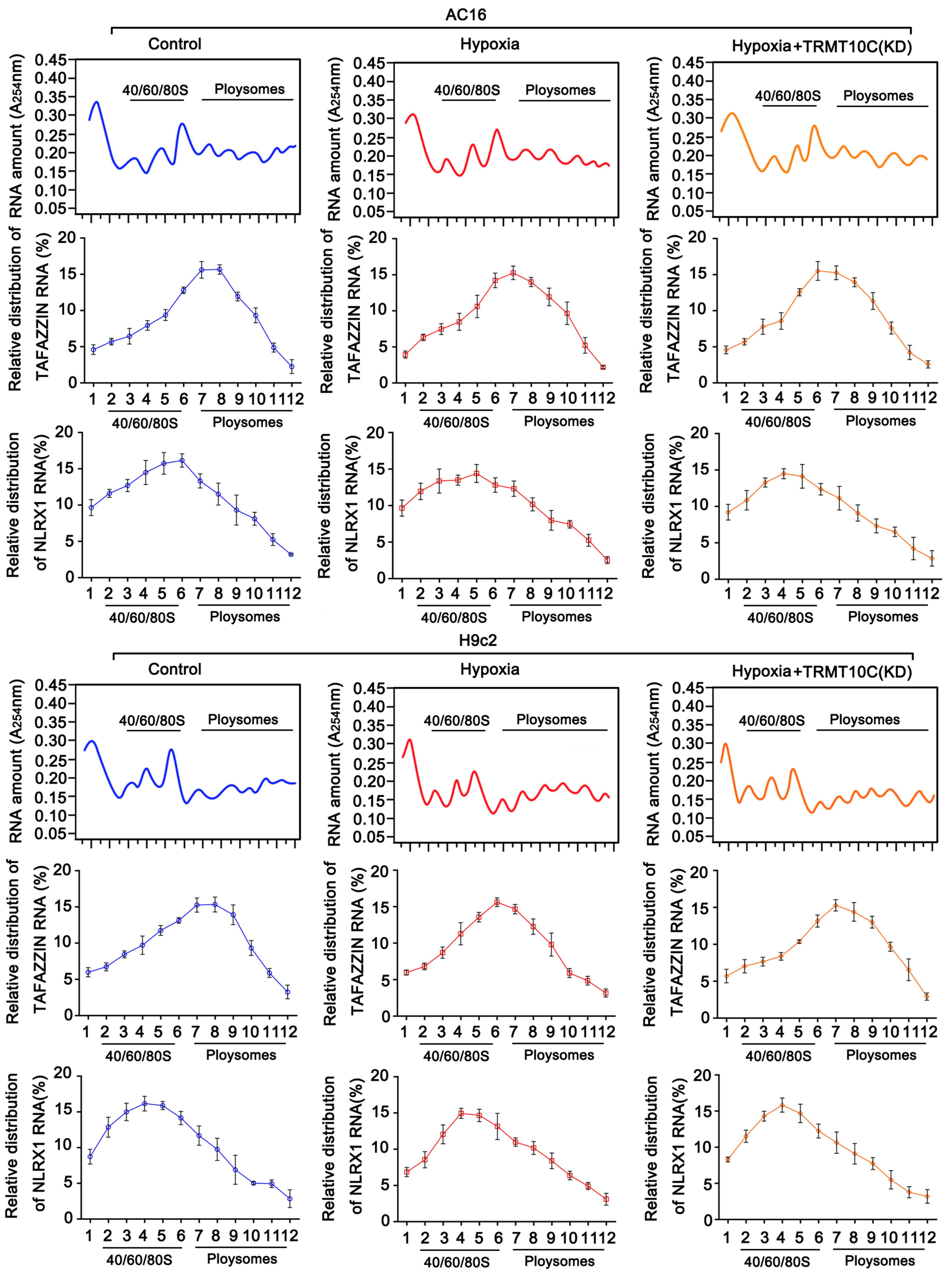

Supplementary Figure 8. The analysis of mRNA translation by Polysome profiling assay.

TRMT10C was knocked down in AC16 and H9c2 cells under hypoxia. The translation of TAFAZZIN and NLRX1 mRNAs was analyzed by polysome profiling assay. The density gradient fractionation system generates a polysome profile, from light to heavy fractions, includes, fractions with 40S, 60S, 80S (monosomes), and

polysomes. Since these peaks did not appear to be different in the treated cells compared to control, the treatment does not seem to have a major effect on the mRNA translation.

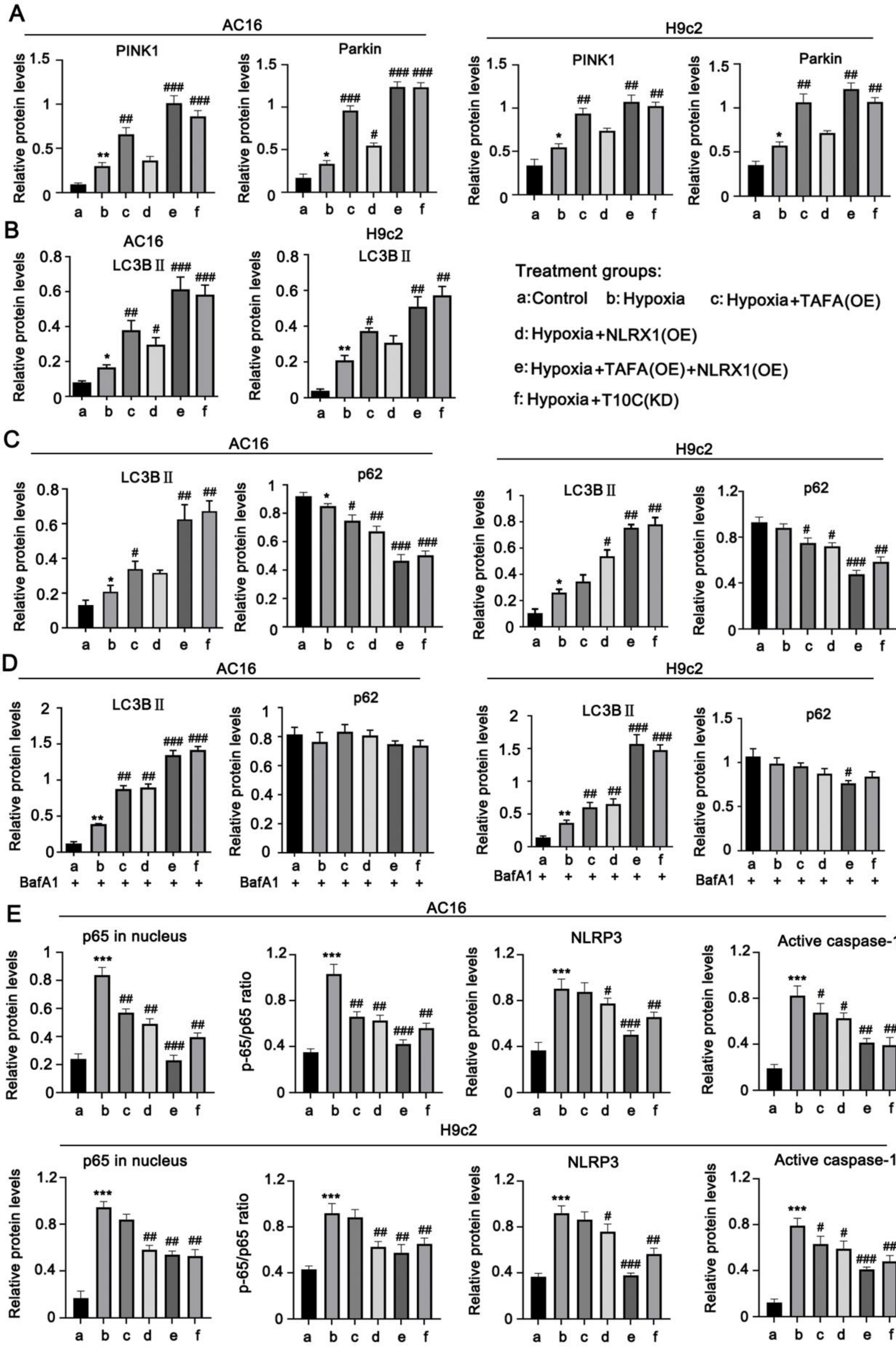

Supplementary Figure 9. Quantitative analysis of protein levels after western blot assay.

A, AC16 and H9c2 cells were transfected with the expression vectors of TAFAZZIN

and NLRX1 to suppress their reduction under hypoxia. Additionally, TRMT10C was knocked down in AC16 and H9c2 cells under hypoxic conditions. Western blotting was conducted to detect the protein levels of PINK1 and Parkin.

B, Western blotting was conducted to detect the protein levels of LC3 in mitochondria.

C,Western blotting was conducted to detect the protein levels of LC3, and p62 in cells.

D, Western blotting was conducted to detect the protein levels of LC3, and p62 in cells after treatments with BafA1.

E, Western blotting was conducted to detect the protein levels of p65, NLRP3 and active caspase-1. TAFA:TAFAZZIN. $^{*}p < 0.05$, $^{**}p < 0.01$ and $^{***}p < 0.001$ vs. Control (n = 3); $^{\#}p < 0.05$, $^{\#\#}p < 0.01$ and $^{\#\#\#}p < 0.001$ vs. Hypoxia group (n = 3).

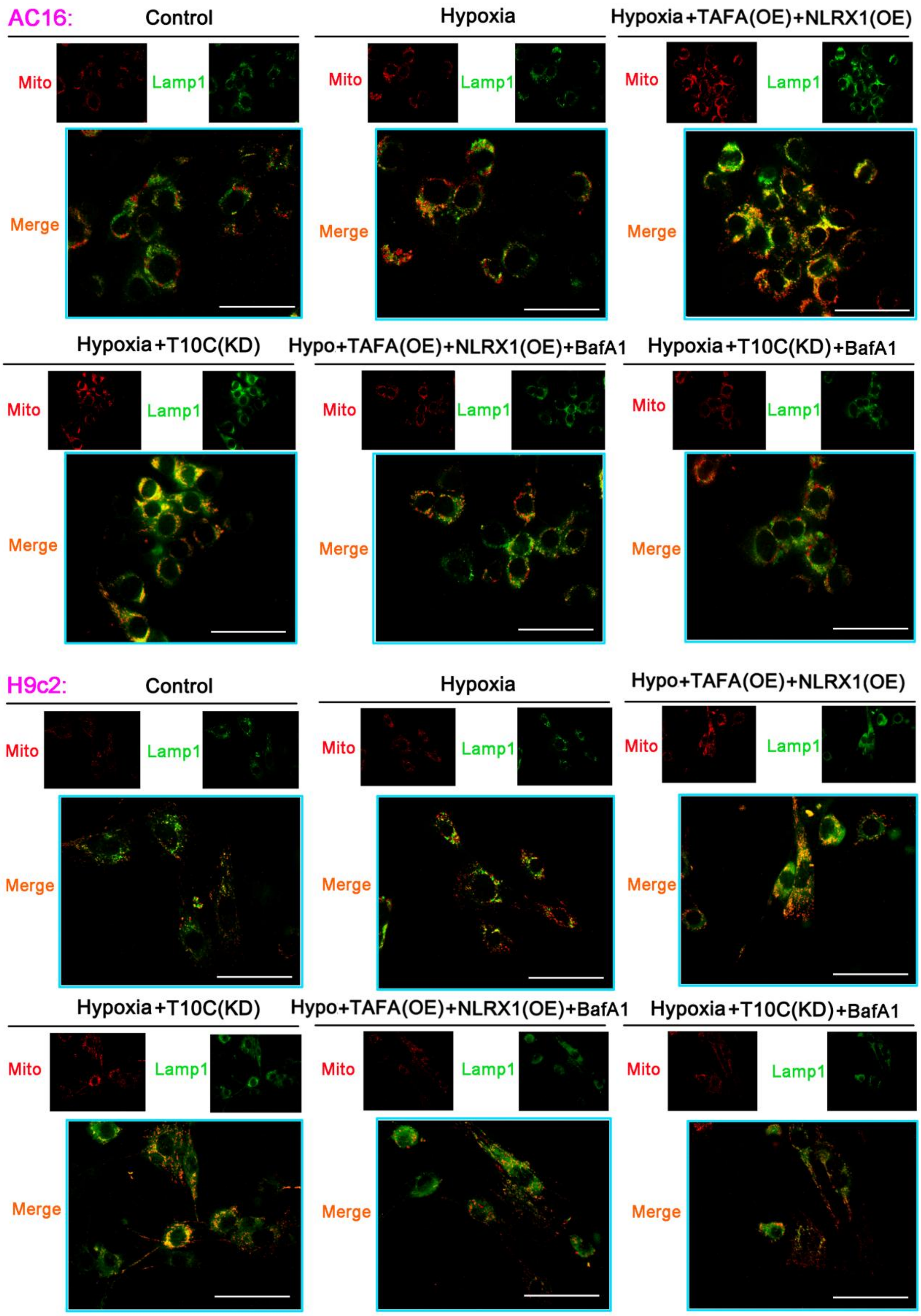

Supplementary Figure 10. The colocalization of mitochondria and lysosome

The interaction between mitochondria and lysosome was investigated by MitoMark red probe and anti-Lamp1 antibody via immunofluorescence assay. Lamp1 is a classic mark of lysosome. Hypoxia moderately promoted the overlap of red fluorescence

(mitochondria) and green fluorescence (lysosome), whereas TAFAZZIN and NLRX1 co-overexpression and TRMT10C knockdown strongly promoted the overlap of red and green fluorescence. BafA1 treatment suppressed the overlap of red and green fluorescence induced by TAFAZZIN and NLRX1 co-overexpression and TRMT10C knockdown. The bar in the figures indicates 10 μm.

Supplementary Table 1. The information for siRNAs

Names	Species	SiRNA NO.	Target Seq
TRMT10C	Human	siRNA1	**AGGCAAAGACTTAATTCATTT**
		siRNA2	TGAATGCCTTCCATTAGATAA
		siRNA3	GAGAACATTTGATGGCTTAAA
	Rat	siRNA1	GAGGAACAACGGGACTTCATGTTTC
		siRNA2	**GCAGAGATATGTGTCTTCCAAAGTA**
		siRNA3	CCATGCAGTTTGGACAGCCTTTGGT
KPNA4	Human	siRNA1	GCGGAACATTTGGTTTCAATT
		siRNA2	**GCAGTAATTGATGCCAATCTT**
		siRNA3	GCACAAGTTGTGCAAGTAGTA
	Rat	siRNA1	**GACTCTGATATAGACGGTGATTATA**
		siRNA2	CGAAATCCACCAATTGATGACTTAA
		siRNA3	CCGCAGGAAATCAACAGCAGGTTCA
YTHDF2	Human	siRNA1	CGGTCCATTAATAACTATAAC
		siRNA2	CCACAGGCAAGGCCCAATAAT
		siRNA3	**CTAGAGAACAACGAGAATAAA**
	Rat	siRNA1	**CTCCTACTTACCCAGTTACTACA**
		siRNA2	CTCTGGATATAGTAGCAATTATG
		siRNA3	GAGAACAACGAGAATAAACCAGT
TAFAZZIN	Human	siRNA1	**TGCTTCCTCAGTTACACAAAG**
		siRNA2	CGGACTTCATTCAAGAGGAAT
		siRNA3	CTTTCCTTCTTGCCTTCAGAT
	Rat	siRNA1	TGGACCAAGTATATGAACCACCTTA
		siRNA2	**CCGTGTACAACAAGGAAGTGCTGTA**
		siRNA3	CAGCTTGGGCAAATGTGTGCCTGTA
NLRX1	Human	siRNA1	AGACCCTTACAAGCATCTATA
		siRNA2	**GCATGACCAGTGCCAAATTAC**
		siRNA3	CGTCAACCTGCTGCGCAAATA
	Rat	siRNA1	GGGCCTTTATCCGTCACCATGGAAA
		siRNA2	GGTGAAATCTGTGGCTTCTCGGATA
		siRNA3	**CAACTTATCCCTGATGTCCTATGCA**
YTHDF3	Human	siRNA1	**TAAGTCAAAGAAGACGTATTA**
		siRNA2	GATAAGTGGAAGGGCAAATTT
		siRNA3	ATAACCAATTACGGCATATTC
	Rat	siRNA1	GAGCCATACTTAAGTAGCCAGACAA
		siRNA2	**CAGTTACGGCTATCCACCTAGTTCT**
		siRNA3	CAGCAGTGGTATGACTAGCATTGCA

Note: The sequences marked in bold were used for formal knockdown experiment.

Supplementary Table 2. The information of primary anti-bodies

Names	Reactivity	Company	Catalogs	Dilution
Succ(K)	Human, Rat, Mouse	PTM Biolabs	PTM-401	1:50
KAT2A	Human, Rat, Mouse	Abcam	ab217876	1:100
CPT1A	Human, Rat, Mouse	Abcam	ab234111	1:100
SIRT5	Human, Rat, Mouse	Abcam	ab259967	1:100
SIRT7	Human, Rat, Mouse	Abcam	ab259968	1:100
Flag	Human, Rat, Mouse	Abcam	ab205606	1:200
His	Human, Rat, Mouse	Abcam	ab18184	1:200
Myc	Human, Rat, Mouse	Abcam	ab9106	1:200
TRMT10C	Human, Rat, Mouse	Santa Cruz	sc-515289	1:100
GAPDH	Human, Rat, Mouse	Proteintech	60004-1-Ig	1:200
COX IV	Human, Rat, Mouse	abcam	ab202554	1:200
Lamin A	Human, Rat, Mouse	Proteintech	81042-1-RR	1:200
β-actin	Human, Rat, Mouse	Proteintech	81115-1-RR	1:200
KPNA2	Human, Rat, Mouse	abcam	ab289858	1:100
KPNA3	Human, Rat, Mouse	Proteintech	67892-1-Ig	1:100
KPNA4	Human, Rat, Mouse	abcam	ab302556	1:100
TAFAZZIN	Human, Rat, Mouse	abcam	ab307148	1:200
NLRX1	Human, Rat, Mouse	Proteintech	17215-1-AP	1:100
PINK1	Human, Rat, Mouse	Proteintech	23274-1-AP	1:50
Parkin	Human, Rat, Mouse	Proteintech	14060-1-AP	1:50
LC3B	Human, Rat, Mouse	abcam	ab192890	1:50
p62	Human, Rat, Mouse	Santa Cruz	sc-48402	1:100
Lamp1	Human, Rat, Mouse	abcam	ab62562	1:50
p65	Human, Rat, Mouse	abcam	ab16502	1:100
p-p65	Human, Rat, Mouse	abcam	ab76302	1:100
NLRP3	Human, Rat, Mouse	abcam	ab263899	1:50
ASC	Human, Rat, Mouse	abcam	ab309497	1:100
Caspase-1	Human, Rat, Mouse	abcam	ab179515	1:50
YTHDF2	Human, Rat, Mouse	abcam	ab220163	1:100

Supplementary Table 3. The information of primers for PCR assay

Names	Species	Direction	Sequence (5' -> 3')	Tm(°C)
TRMT10C	Human	Forward	TCAAGCTGCTAGAAACCACTG	60
		Reverse	TCTGTGCAAAGCACCATCTATT	60
	Rat	Forward	GGAGAGGAACAACGGGACTT	59.3
		Reverse	CTGTCCAAACTGCATGGCCT	60.9
	Mouse	Forward	TGTCCTCCAAAGCACCTTCTT	61.3
		Reverse	TGAATGCTCGACTTCATTGTAGC	60.9
TAFAZZIN	Human	Forward	CACCGTGTCCAATCACCAGTC	62.9
		Reverse	TCCAACGCATCAACTTCAGGT	62
	Rat	Forward	CTGCGACCCCTCTTATCACC	59.8
		Reverse	AAGTCTGTGAGGGCTTTCCG	59.9
	Mouse	Forward	ATGCCCCTCCATGTGAAGTG	61.9
		Reverse	GTGCCAACTAGGCCCATGAC	62.9
NLRX1	Human	Forward	ACGGGACTTTGTAGTGACCC	61.2
		Reverse	CCTGAGGCAGCATGTATTTGC	61.7
	Rat	Forward	AGCCCGGACTATGGGTAAGT	60
		Reverse	TGACATCTTCCCCAACACGG	59.9
	Mouse	Forward	TAGGGCCTTTATCCGTTACCA	60
		Reverse	TAAACCACTCGGTGAGGTTCC	61.4
